# RIPK3 promotes brain region-specific interferon signaling and restriction of tick-borne flavivirus infection

**DOI:** 10.1371/journal.ppat.1011813

**Published:** 2023-11-27

**Authors:** Marissa Lindman, Juan P. Angel, Irving Estevez, Nydia P. Chang, Tsui-Wen Chou, Micheal McCourt, Colm Atkins, Brian P. Daniels

**Affiliations:** Department of Cell Biology and Neuroscience, Rutgers University, Piscataway, New Jersey, United States of America; University of Iowa, UNITED STATES

## Abstract

Innate immune signaling in the central nervous system (CNS) exhibits many remarkable specializations that vary across cell types and CNS regions. In the setting of neuroinvasive flavivirus infection, neurons employ the immunologic kinase receptor-interacting kinase 3 (RIPK3) to promote an antiviral transcriptional program, independently of the traditional function of this enzyme in promoting necroptotic cell death. However, while recent work has established roles for neuronal RIPK3 signaling in controlling mosquito-borne flavivirus infections, including West Nile virus and Zika virus, functions for RIPK3 signaling in the CNS during tick-borne flavivirus infection have not yet been explored. Here, we use a model of Langat virus (LGTV) encephalitis to show that RIPK3 signaling is specifically required in neurons of the cerebellum to control LGTV replication and restrict disease pathogenesis. This effect did not require the necroptotic executioner molecule mixed lineage kinase domain like protein (MLKL), a finding similar to previous observations in models of mosquito-borne flavivirus infection. However, control of LGTV infection required a unique, region-specific dependence on RIPK3 to promote expression of key antiviral interferon-stimulated genes (ISG) in the cerebellum. This RIPK3-mediated potentiation of ISG expression was associated with robust cell-intrinsic restriction of LGTV replication in cerebellar granule cell neurons. These findings further illuminate the complex roles of RIPK3 signaling in the coordination of neuroimmune responses to viral infection, as well as provide new insight into the mechanisms of region-specific innate immune signaling in the CNS.

## Author summary

Interactions between the nervous and immune systems are very carefully orchestrated in order to protect the brain and spinal cord from immune-mediated damage, while still maintaining protective defenses against infection. These specialized neuro-immune interactions have been shown to vary significantly across regions of the brain, with innate antiviral signaling being particularly strong in the cerebellum, although the reasons for this are poorly understood. Here, we show a specialized adaptation of programmed cell death signaling that uniquely protects the cerebellum from tick-borne flavivirus infection. These findings provide important new insight into the molecular mechanisms that promote the uniquely robust antiviral immunity of the cerebellum. They also provide new clues into the pathogenesis of tick-borne encephalitis, a zoonosis of significant global concern.

## Introduction

Flaviviruses are a family of positive sense RNA viruses which include several notable pathogens associated with neuroinvasive infection in humans, including West Nile virus (WNV), Zika virus (ZIKV), and Japanese Encephalitis virus [1]. While nearly all major flaviviruses are transmitted by mosquito vectors, a small but significant number of flaviviruses are transmitted by ticks, including Tick-borne encephalitis virus (TBEV) and its close relatives that together make up a single TBEV serocomplex. Tick borne encephalitis is a significant and growing threat to public health, particularly in Europe and northern Asia, where TBEV constitutes the most prevalent tick-borne zoonotic disease [2–4]. Notably, some TBEV strains elicit mortality rates up to 40% in humans [5], underscoring the urgent need to better understand the mechanisms underlying the pathogenesis of tick-borne flavivirus infections.

Effective control of flavivirus infection in the central nervous system (CNS) requires robust innate immune signaling in neural cells, particularly neurons, which are the predominantly infected cell type in most cases of flavivirus encephalitis [6–9]. Effective type I interferon (IFN) signaling is of particular importance for innate control of viral replication in neurons [10–12]. Notably, differences in type I IFN signaling across neural cell types and brain regions are associated with differential susceptibility to flavivirus infection. For example, previous reports suggest that the enhanced type I IFN signaling observed in hindbrain regions compared to the forebrain is an underlying determinant of the enhanced susceptibility of forebrain regions to WNV infection [12,13]. However, the unique signaling mechanisms that promote differential IFN-mediated control of viral infection in the hindbrain have not been extensively characterized.

A potential regulator of neuronal IFN signaling during flavivirus infection is receptor interacting protein kinase-3 (RIPK3). RIPK3 is an enzyme traditionally associated with necroptosis, a form of lytic programmed cell death [14]. Necroptosis occurs via the RIPK3-dependent activation of mixed lineage kinase domain like protein (MLKL), which forms oligomeric pore complexes that induce cellular lysis [15]. However, many recent studies have identified complex roles for RIPK3 signaling in the coordination of inflammation, including the regulation of inflammatory transcriptional responses that occur independently of necroptosis [16–24]. We and others have demonstrated that RIPK3 signaling in neurons is of particular importance for the control of neurotropic viral infections, as neuronal RIPK3 promotes a robust antimicrobial transcriptional program, including many IFN stimulated genes (ISGs), that restricts viral infection without inducing neuronal necroptosis [16,17]. Other recent studies have identified unexpected roles for RIPK3 in the regulation of type I IFN signaling, via mechanisms which include the regulation of pattern recognition receptor signaling and protein kinase-R (PKR)-mediated stabilization of *Ifnb* mRNA [18,19].

In this study, we interrogated roles for RIPK3 in controlling tick-borne flavivirus infection. To do so, we used Langat virus (LGTV), a naturally attenuated member of the TBEV serocomplex that can be studied under BSL2 containment. LGTV infection is neuroinvasive in rodents and results in mild clinical disease [25,26]. *Ripk3*^-/-^ mice exhibited enhanced neurologic disease following subcutaneous LGTV infection, while *Mlkl*^-/-^ mice were indistinguishable from littermate controls, suggesting a necroptosis-independent function for RIPK3 in restricting LGTV pathogenesis. Notably, *Ripk3*^-/-^ mice exhibited increased viral burden in the cerebellum, along with diminished expression of inflammatory chemokines and ISGs in the cerebellum, but not the cerebral cortex. *In vitro* analysis of cultured primary cortical and cerebellar cell types showed that pharmacologic inhibition of RIPK3 resulted in enhanced LGTV replication in cerebellar granule cell neurons but not in cortical neurons or in astrocytes and microglia derived from either brain region. Transcriptional profiling showed that RIPK3 signaling was uniquely required for the full induction of ISG expression in cerebellar granule cell neurons, demonstrating a previously unknown, region-specific function for RIPK3 in coordinating innate antiviral immunity within the CNS.

## Results

### RIPK3 controls LGTV pathogenesis independently of MLKL and peripheral immunity

**Fig 1:**
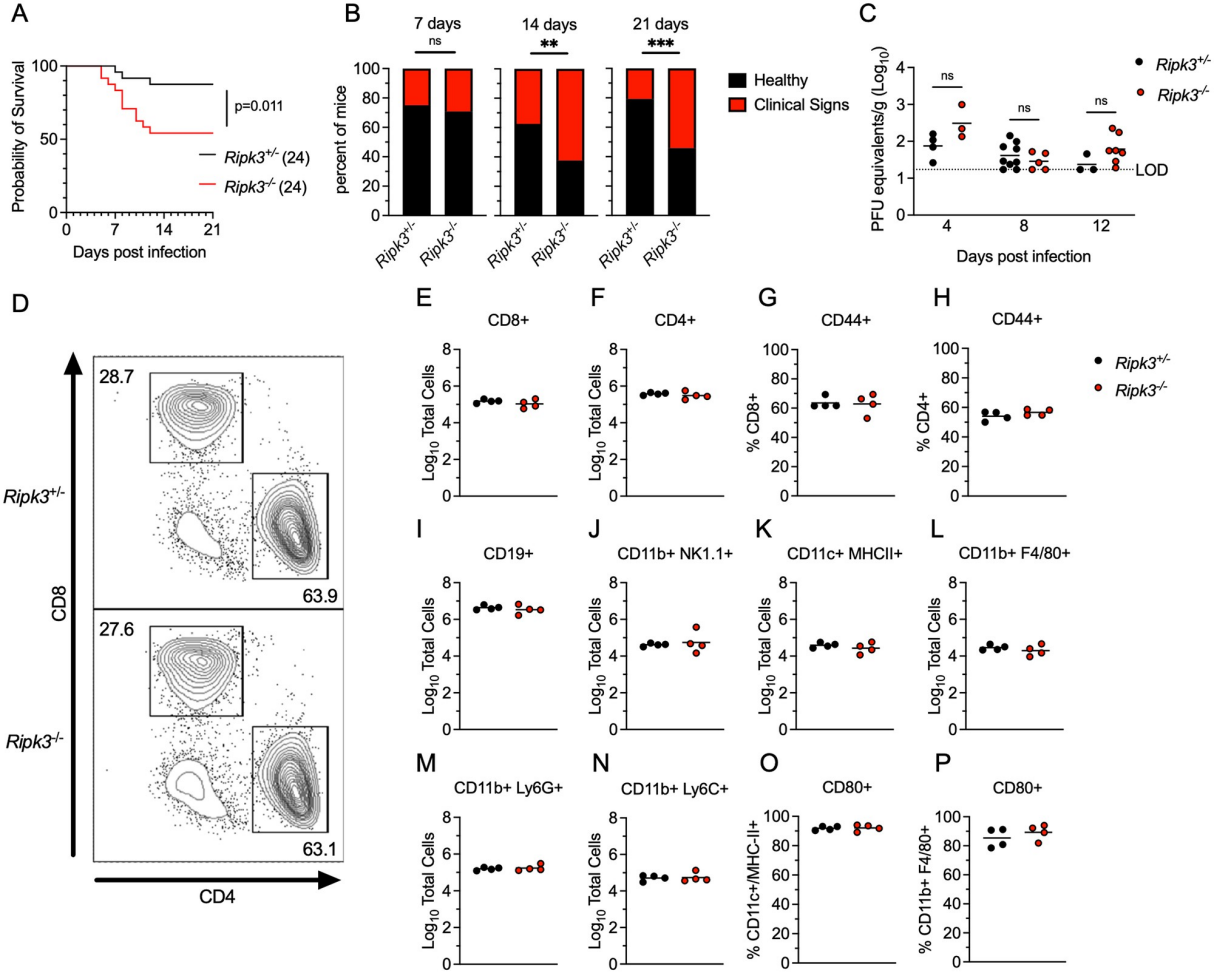
RIPK3 limits LGTV pathogenesis independently of peripheral immunity. (A-B) Survival analysis (A) and presentation of clinical signs of disease (B) in *Ripk3*^-/-^ mice and littermate controls following subcutaneous inoculation with 3x10^4^ PFU LGTV TP21. Data are pooled from two experiments. (C) *Ripk3*^-/-^ and littermate control mice were infected subcutaneously with LGTV TP21. On indicated days following infection, splenic viral burden was measured via qRT-PCR. Data was normalized against a standard curve of known viral titers to generate plaque-forming unit (PFU) equivalents. Data for each day post infection are pooled from 2–3 experiments. LOD, limit of detection. (D-P) *Ripk3*^-/-^ and littermate control mice were infected subcutaneously with LGTV TP21 for 8 days prior to harvesting splenocytes and profiling leukocytes by flow cytometry. (D) Representative flow cytometry plots showing CD8+ and CD4+ T cells among CD3+ leukocytes in the spleen. Numbers represent percentage of cells in each gate relative to total plotted cells. (E-F) Numbers of CD8+ T cells (E) and CD4+ T cells (F) among CD3+ leukocytes. (G-H) Percentage of CD44+ cells among CD8+ T cells (G) and CD4+ T cells (H). (I-N) Numbers of CD19+ B cells (I), CD11b+ NK1.1+ Natural Killer cells (J), CD11c+ MHC-II+ dendritic cells (K), CD45high CD11b+ F4/80+ macrophages (L), CD11b+ Ly6G+ neutrophils (M), and CD45high CD11b+ Ly6C+ monocytes (N) among total leukocytes in the spleen. (O-P) Percentage of CD80+ cells among CD11c+ MHC-II+ dendritic cells (O) and CD11b+ F4/80+ macrophages (P). ns, not significant. **p < 0.01, ***p < 0.001.

To assess the role of RIPK3 in controlling LGTV pathogenesis, we subcutaneously infected *Ripk3*^-/-^ mice, along with heterozygous littermate controls, with 3x10^4^ plaque forming units (pfu) of the Malaysian LGTV strain TP21. We note that *Ripk3*^+/-^ animals do not exhibit haploinsufficiency and are routinely used as littermate controls in studies of this pathway [27–29]. Control animals exhibited limited mortality following LGTV infection (Fig 1A), consistent with previous reports [25,26]. However, mice lacking *Ripk3* expression exhibited a significantly accelerated and enhanced rate of mortality (Fig 1A). In addition, a higher proportion of *Ripk3*^*-/-*^ mice exhibited clinical signs of neurologic disease, including paresis or full hindlimb paralysis, by 14 days post infection (dpi) (Fig 1B), and this difference persisted to at least 21 dpi. These data suggest that *Ripk3* is essential for restricting neuropathogenesis during LGTV infection.

To better understand this phenotype, we first assessed whether RIPK3 was required for early control of systemic infection. Spleens of infected mice exhibited low levels of LGTV RNA that were not impacted by *Ripk3* expression (Fig 1C). To test whether *Ripk3*^-/-^ mice exhibited any deficiencies in peripheral immune responses, we performed flow cytometric analysis of major immune cell subsets in the spleens of infected animals at 8 dpi. *Ripk3*^-/-^ animals exhibited similar frequencies (Fig 1D) and total numbers (Fig 1E and 1F) of CD4 and CD8 T cells among all splenocytes compared to littermate controls, as well as similar rates of CD44 expression (a key T cell activation marker) across both subsets (Fig 1G and 1H). Numbers of B cells (Fig 1I) and natural killer (NK) cells (Fig 1J) were also similar between genotypes. In the myeloid compartment, we observed similar numbers of CD11c^+^ MHCII^+^ dendritic cells (Fig 1K) between genotypes, as well as similar numbers of myeloid subsets expressing F4/80 (Fig 1L), Ly6G (Fig 1M), and Ly6C (Fig 1N). Both CD11c^+^ MHCII^+^ and F4/80^+^ antigen presenting cell subsets also exhibited similar rates of expression of the costimulation signal CD80 between genotypes (Fig 1O and 1P). These data suggest that *Ripk3*^-/-^ mice mounted normal peripheral immune responses to subcutaneous LGTV challenge, similar to our previous observations with WNV and ZIKV [16,17]. Thus, the increased pathogenesis observed in mice lacking *Ripk3* was unlikely to arise from a failure in peripheral virologic control.

**Fig 2:**
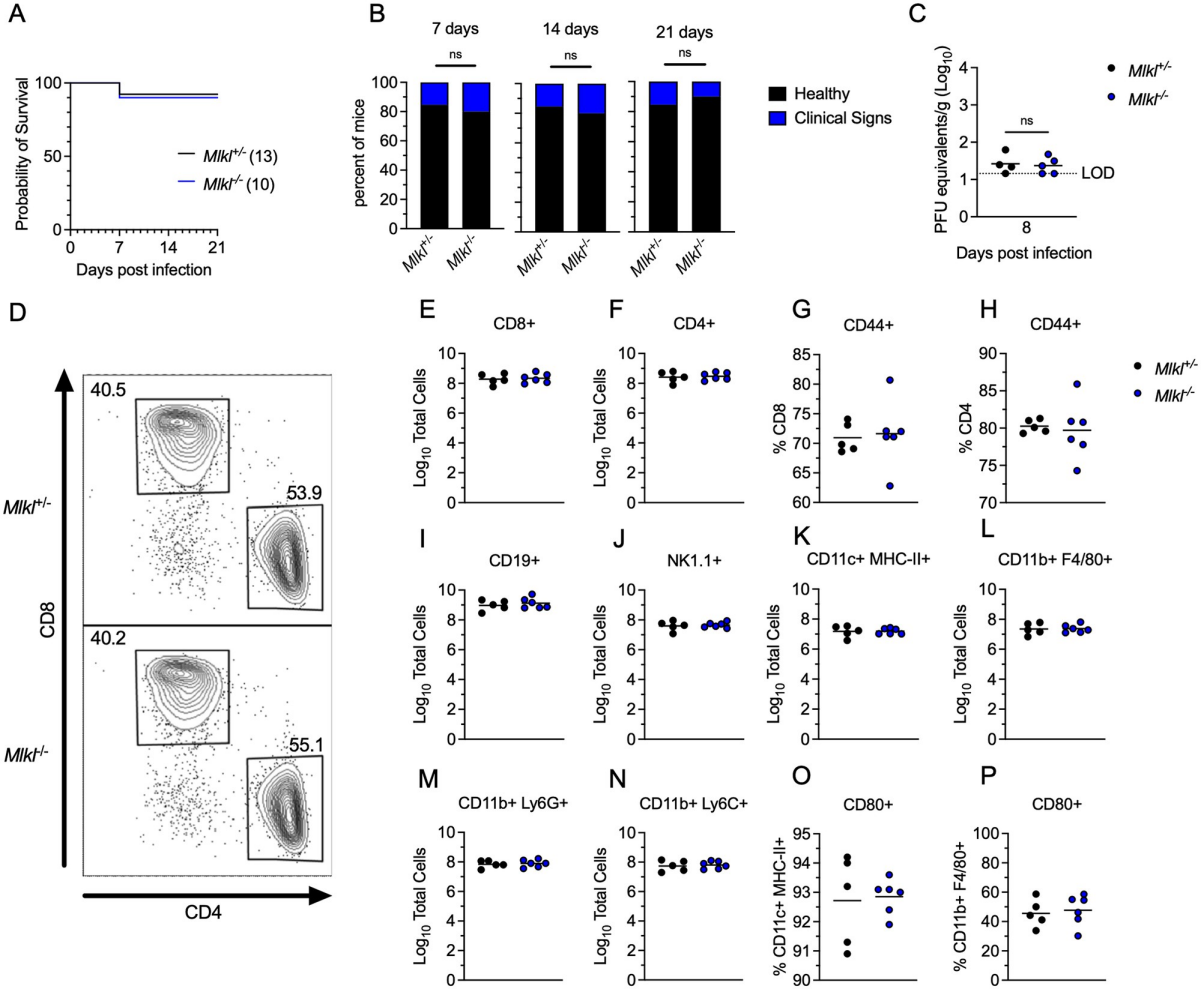
MLKL signaling does not influence Langat virus pathogenesis. (A-B) Survival analysis (A) and presentation of clinical signs of disease (B) in *Mlkl*^-/-^ mice and littermate controls following subcutaneous inoculation with 3X10^4^ PFU LGTV TP21. Data are pooled from two experiments. (C) *Mlkl*^-/-^ and littermate control mice were infected subcutaneously with LGTV TP21. On indicated days following infection, splenic viral burden was measured via qRT-PCR. Data was normalized against a standard curve of known viral titers to generate plaque-forming unit (PFU) equivalents. Data for each day post infection are pooled from 2–3 experiments. LOD, limit of detection. (D-P) *Mlkl*^-/-^ and littermate control mice were infected subcutaneously with LGTV TP21 for 8 days prior to harvesting splenocytes and profiling leukocytes by flow cytometry. (D) Representative flow cytometry plots showing CD8+ and CD4+ T cells among CD3+ leukocytes in the spleen. Numbers represent percentage of cells in each gate relative to total plotted cells. (E-F) Numbers of CD8+ T cells (E) and CD4+ T cells (F) among CD3+ leukocytes. (G-H) Percentage of CD44+ cells among CD8+ T cells (G) and CD4+ T cells (H). (I-N) Numbers of CD19+ B cells (I), CD11b+ NK1.1+ Natural Killer cells (J), CD11c+ MHC-II+ dendritic cells (K), CD45high CD11b+ F4/80+ macrophages (L), CD11b+ Ly6G+ neutrophils (M), and CD45high CD11b+ Ly6C+ monocytes (N) among total leukocytes in the spleen. (O-P) Percentage of CD80+ cells among CD11c+ MHC-II+ dendritic cells (O) and CD11b+ F4/80+ macrophages (P). ns, not significant.

A potential mechanism by which RIPK3 signaling might restrict LGTV pathogenesis is through the induction of necroptosis in infected cells. We thus tested whether loss of the necroptotic executioner molecule MLKL would impact disease course following subcutaneous LGTV infection. Notably, *Mlkl*^-/-^ mice exhibited no difference in either survival or development of clinical disease signs compared to littermate controls (Fig 2A and 2B). We saw similarly that *Mlkl*^-/-^ did not exhibit altered splenic viral burden at 8dpi (Fig 2C). Flow cytometric analysis also revealed essentially identical numbers and frequencies of all major immune cell subsets in the spleen at this time point (Fig 2C–2P). Multistep growth curve analysis also demonstrated that neither RIPK3 nor MLKL impacted the low levels of LGTV replication observed in primary leukocyte cultures, including bone marrow derived macrophages and dendritic cells (S1A and S1B Fig). These data suggest that MLKL, and therefore necroptosis, is not a major contributor to peripheral virologic control or overall disease pathogenesis in the setting of LGTV infection, and thus that RIPK3 exerts its protective effect in this model through an alternative mechanism.

### RIPK3 is required for CNS-intrinsic restriction of LGTV infection

**Fig 3:**
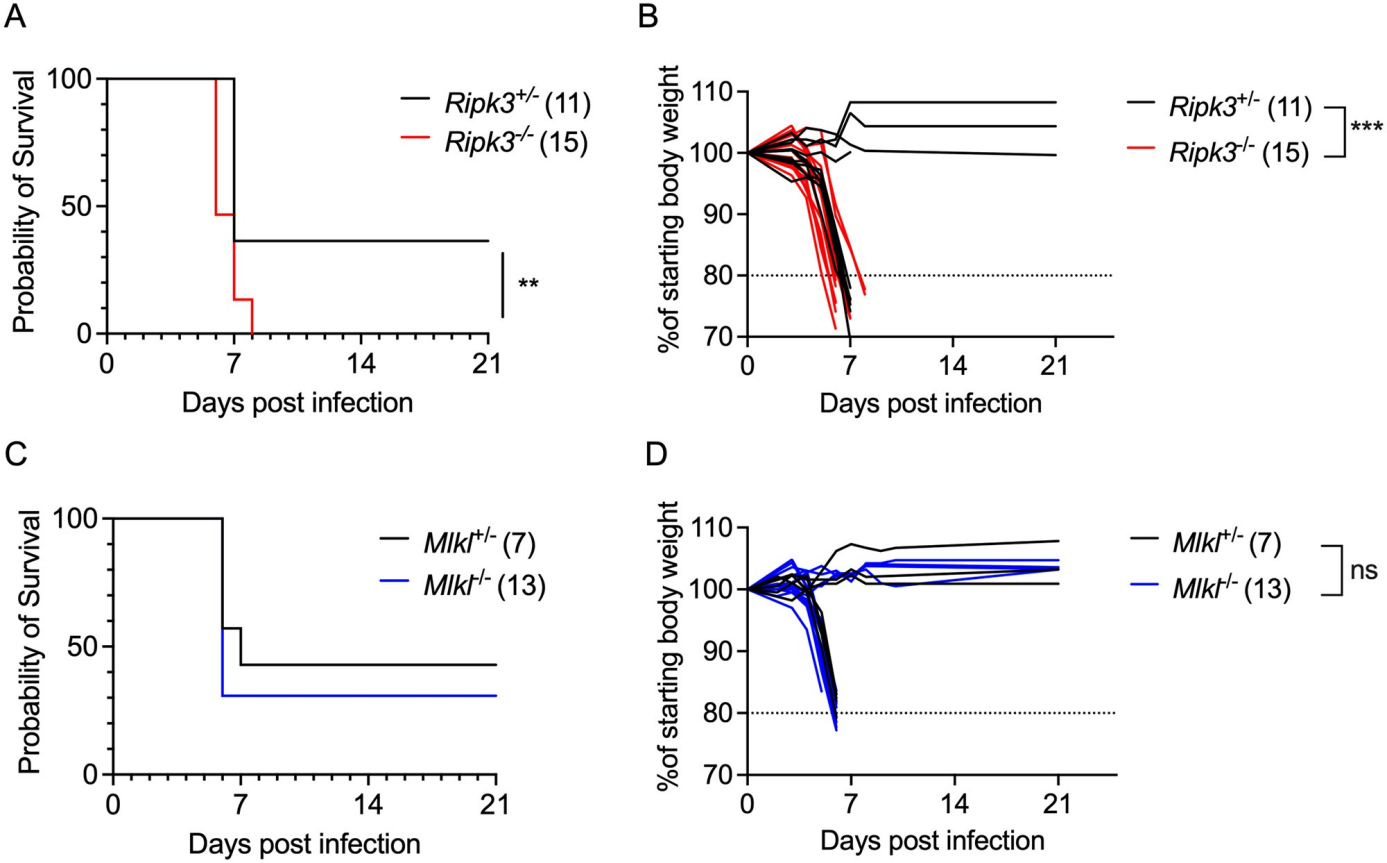
RIPK3, but not MLKL, restricts Langat virus pathogenesis following intracranial infection. Survival and body weight analysis from *Ripk3*^-/-^ (A-B) and *Mlkl*^-/-^ (C-D) mice and their respective littermate controls following intracranial inoculation with 50 PFU LGTV TP21. Data are pooled from two (A-B) or three (C-D) experiments. ns, not significant. **p < 0.01, ***p < 0.001.

Because we did not observe differences in peripheral virologic control in *Ripk3*^-/-^ mice, we next questioned whether RIPK3 acted in a CNS-intrinsic manner to limit LGTV infection. To assess this, we next used an intracranial infection route in order to assess local effects of RIPK3 signaling on LGTV pathogenesis. *Ripk3*^-/-^ mice exhibited accelerated and enhanced mortality compared to littermate controls following intracranial infection (Fig 3A). *Ripk3*-deficient mice also exhibited worsened clinical disease prior to death, as evidenced by earlier and more dramatic weight loss following infection (Fig 3B). In contrast, *Mlkl*^-/-^ mice were indistinguishable from littermate controls in terms of overall mortality (Fig 3C) and weight loss (Fig 3D) following intracranial infection. These data further supported the idea that RIPK3 restricts LGTV neuropathogenesis via CNS-intrinsic mechanisms, independently of necroptosis.

### RIPK3 promotes neuronal chemokine expression in a region-specific manner following LGTV infection

**Fig 4:**
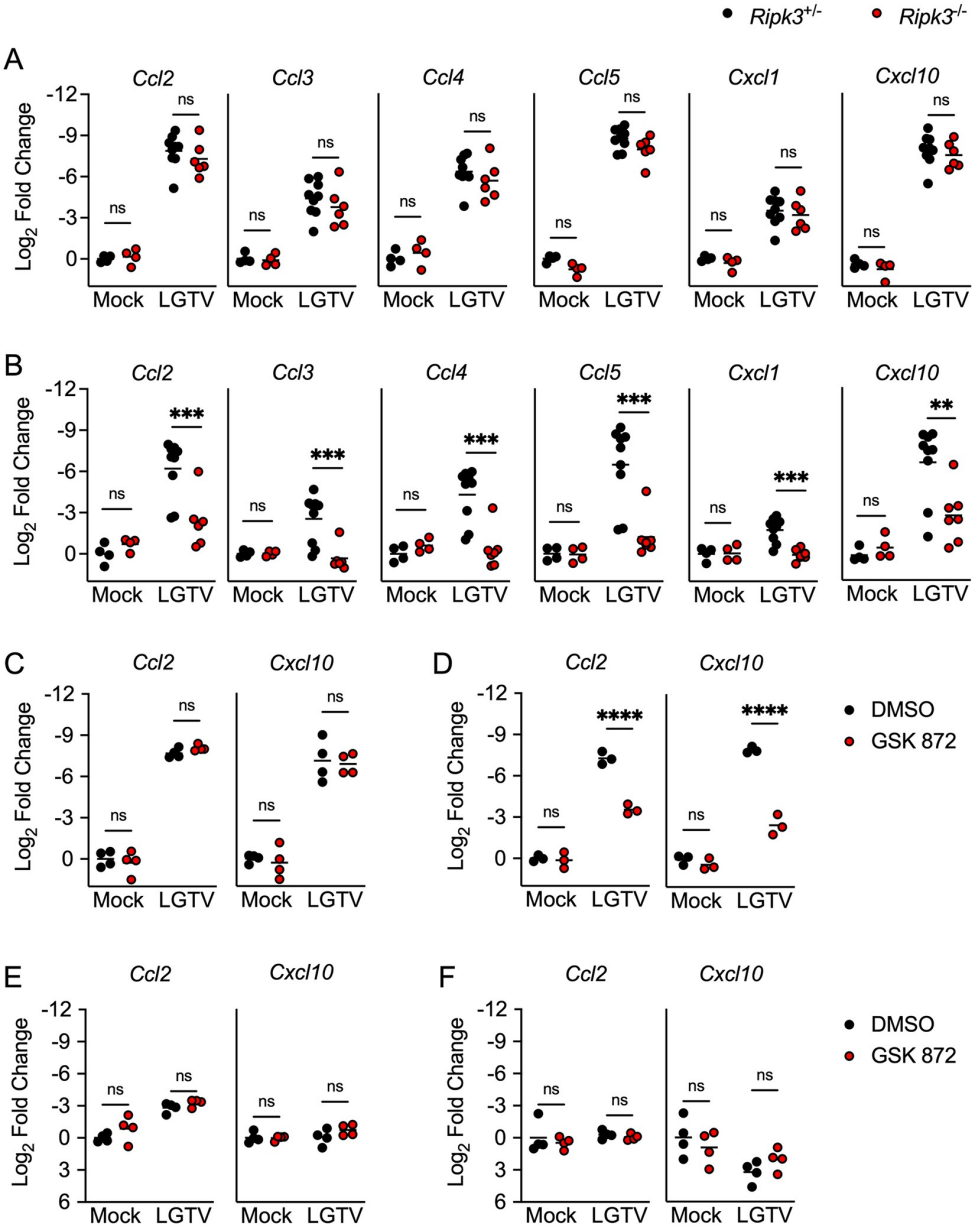
RIPK3 promotes chemokine expression in the cerebellum during LGTV encephalitis. (A-B) *Ripk3* ^-/-^ and littermate control mice were infected subcutaneously with LGTV TP21. At 8dpi cerebral cortical (A) and cerebellar tissues (B) were harvested and assayed for chemokine transcripts via qRT-PCR. (C-F) *Ccl2* and *Cxcl10* expression in wildtype (C57BL/6J) cultures of primary cortical neurons (C), cerebellar granule cell neurons (D), cortical astrocytes (E), and cerebellar astrocytes (F) following 2-hour pretreatment with GSK 872 or vehicle and 24h infection with 0.5 (C-D) or 0.01 (E-F) MOI LGTV TP21, measured via qRT-PCR. ns, not significant. *p<0.05, **p < 0.01, ***p < 0.001, ****p < 0.0001.

We and others previously showed that neuronal RIPK3 signaling was required for the expression of key inflammatory chemokines that served to restrict WNV pathogenesis by coordinating the recruitment of leukocytes into the infected CNS. We thus questioned whether RIPK3 also promotes chemokine expression in the CNS during LGTV infection. Surprisingly, transcriptional profiling in the cerebral cortex of *Ripk3*^-/-^ mice following subcutaneous LGTV infection revealed no differences in expression of major chemokines compared to littermate controls (Fig 4A). However, we did observe significantly diminished chemokine responses in cerebellar tissues derived from *Ripk3*^-/-^ animals (Fig 4B). We confirmed differential expression of CXCL10 in the cerebella but not cerebral cortices of *Ripk3*^-/-^ mice at the protein level by ELISA (S2A and S2B Fig). To understand which cell types were driving this region-specific deficit in chemokine expression, we next cultured primary neurons and astrocytes derived specifically from either cerebral cortex or cerebellum and infected with LGTV, with or without a small molecule inhibitor of RIPK3 (GSK 872). Consistent with our *in vivo* findings, blockade of RIPK3 in cerebral cortical neurons did not impact chemokine expression following LGTV infection (Fig 4C). In contrast, infected cerebellar granule cell neuron cultures exhibited significantly diminished chemokine expression when RIPK3 was inhibited by GSK 872 (Figs 4D and S2C). Notably, we did not observe a dependence on RIPK3 for the expression of chemokines in astrocytes derived from either region (Fig 4E and 4F). These data suggest that RIPK3 serves an unexpected, region-specific transcriptional function in neurons of the cerebellum during neuroinvasive LGTV infection.

### RIPK3 is not required for immune cell recruitment to the LGTV-infected CNS

**Fig 5:**
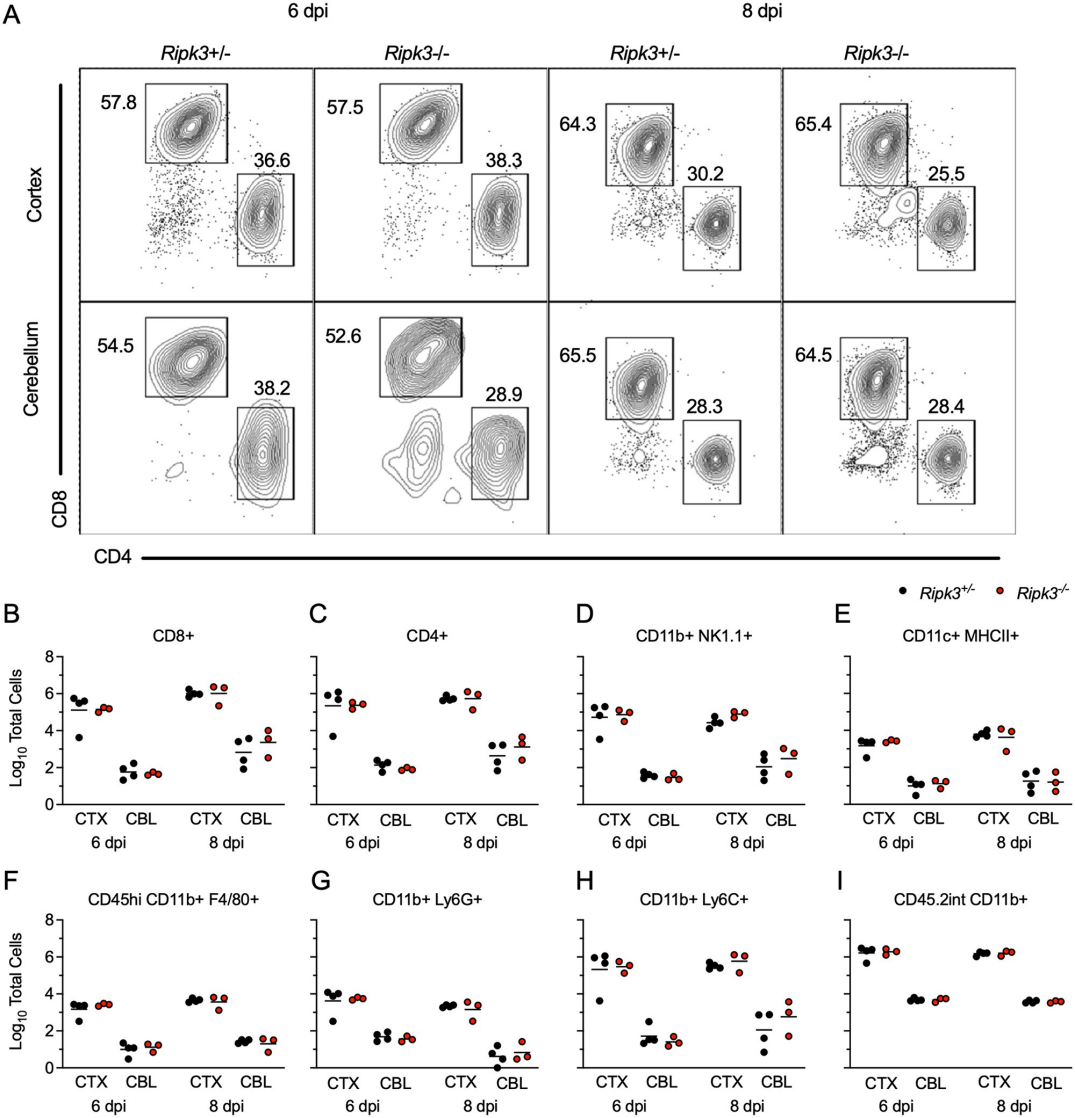
Leukocyte recruitment to the CNS occurs independently of RIPK3 signaling during LGTV encephalitis. (A-I) *Ripk3* ^-/-^ and littermate control mice were infected subcutaneously with LGTV TP21. Cerebral cortical and cerebellar tissues were harvested and leukocytes isolated for flow cytometric profiling at indicated days post infection (dpi). (A) Representative flow cytometry plots showing CD8+ and CD4+ T cells among CD3+ leukocytes in the brain. Numbers represent percentage of cells in each gate relative to total plotted cells. (B-I) Numbers of CD8+ T cells (B), CD4+ T cells (C), CD11b+ NK1.1+ natural killer cells (D), CD11c+ MHC-II+ dendritic cells (E), CD45^high^ CD11b+ F4/80+ macrophages (F), CD11b+ Ly6G+ neutrophils (G), CD45^high^ CD11b+ Ly6C+ monocytes (H), and CD45.2^lo^ CD11b+ microglia (I) among total brain leukocytes. No comparisons are statistically significant.

We next questioned whether diminished chemokine expression in the cerebellum of *Ripk3*^-/-^ mice would result in a failure to recruit antiviral leukocytes into this brain region. We thus performed flow cytometric analysis of leukocytes derived from either cerebral cortex or cerebellum following subcutaneous LGTV infection. Remarkably, we saw no evidence of changes in lymphocyte recruitment in either brain region of *Ripk3*^-/-^ mice compared to littermate controls on either 6 or 8 dpi (Fig 5A). This lack of difference extended across all major CD45^hi^ infiltrating leukocyte subsets, including CD4^+^ and CD8^+^ T cells (Fig 5B and 5C), NK cells (Fig 5D), CD11c^+^ MHCII^+^ dendritic cells (Fig 5E) and myeloid subsets expressing F4/80 (Fig 5F), Ly6G (Fig 5G), and Ly6C (Fig 5H). We similarly did not observe differences in numbers of CD45^lo^ microglia (Fig 5I), suggesting no major differences in microglial proliferation between genotypes in either region. These data suggested that, despite significant differences in the expression of major leukocyte chemoattractants in the cerebellum, differences in immune cell recruitment did not account for the increased pathogenesis observed in *Ripk3*^-/-^ mice during LGTV infection.

### RIPK3 promotes cell-intrinsic restriction of LGTV replication in cerebellar neurons

**Fig 6:**
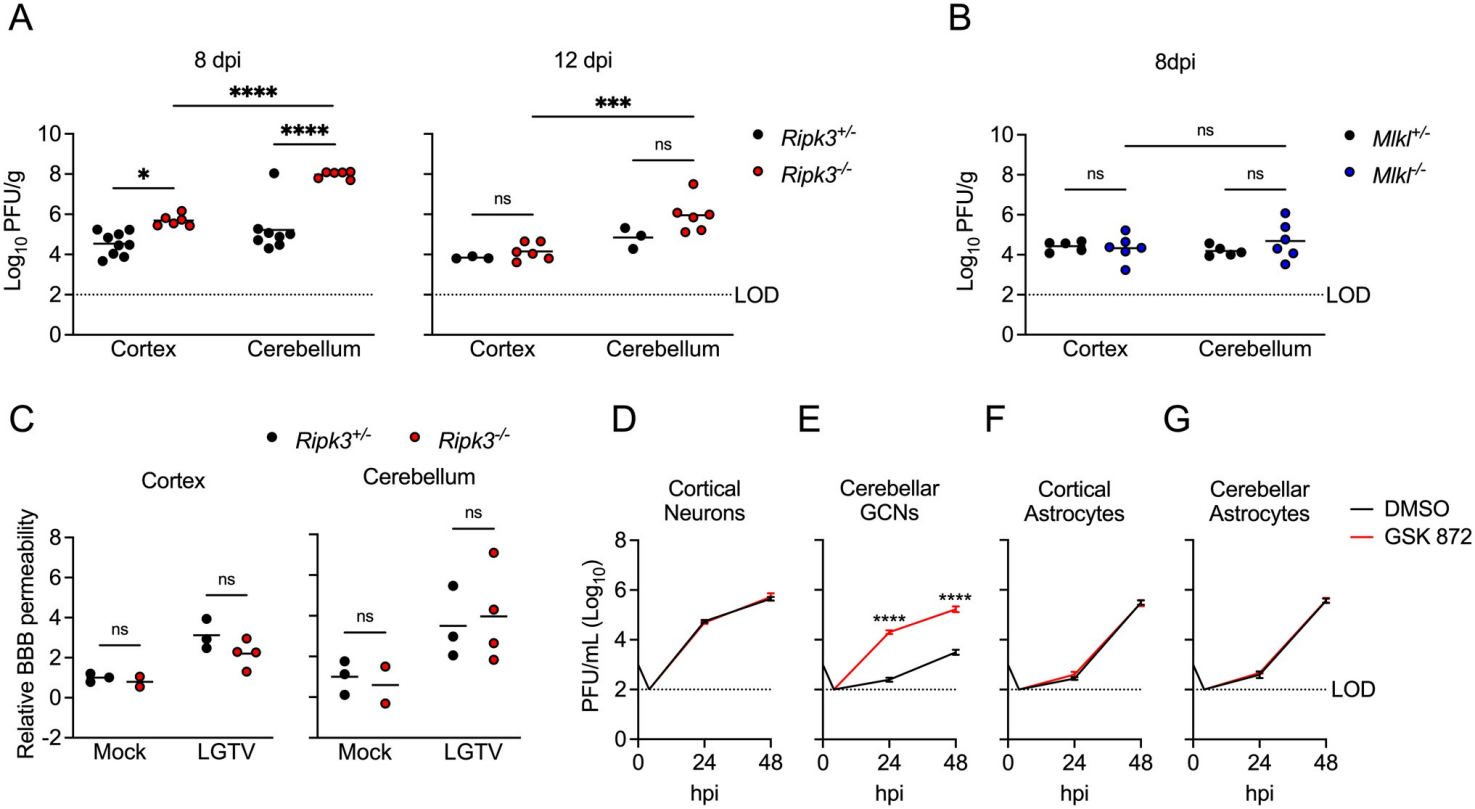
RIPK3 limits LGTV replication in cerebellar granule cell neurons. (A-B) *Ripk3* ^-/-^ (A) or *Mlkl* ^-/-^ (B) mice and littermate controls were infected subcutaneously with LGTV TP21. At 8 or 12 days post infection (dpi), viral loads in cerebral cortical and cerebellar tissues were determined by plaque assay. Data are pooled from 2–3 independent experiments. (C) *Ripk3* ^-/-^ and littermate control mice were subcutaneously infected with LGTV TP21. BBB permeability was measured at 8 dpi by detection of sodium fluorescein accumulation in tissue homogenates derived from cerebral cortex or cerebellum. Data represent individual brain fluorescence values normalized to serum sodium fluorescein concentration. Individual mouse values were then normalized to the mean values for uninfected controls. (D-G) Multistep growth curve analysis following infection with 0.01 MOI LGTV TP21 in cortical neurons (D), cerebellar granule cell neurons (E), cortical astrocytes (F), and cerebellar astrocytes (G). n = 3 (cerebellar granule cell neurons) or 4 (astrocytes and cortical neurons) for growth curve experiments. ns, not significant. *p<0.05, **p < 0.01, ***p < 0.001, ****p < 0.0001.

Given these observations, we next questioned whether *Ripk3*^-/-^ mice fail to control LGTV infection due to impaired innate immune restriction of LGTV replication. Assessment of viral burdens in brains of *Ripk3*^-/-^ mice following subcutaneous LGTV infection revealed that *Ripk3*^-/-^ mice exhibited significantly elevated CNS viral titers, particularly in the cerebellum, at both 8 and 12 dpi (Fig 6A). In contrast, *Mlkl*^-/-^ exhibited no such difference in viral burden in either brain region (Fig 6B). Differences in viral burden did not appear to be linked to deficits in blood-brain barrier integrity, as both *Ripk3*^-/-^ mice and littermate controls exhibited similar levels of sodium fluorescein extravasation into the CNS following infection (Fig 6C). We thus questioned whether RIPK3 was required for cell-intrinsic restriction of viral replication in susceptible CNS cell types. While LGTV preferentially infects neurons *in vivo* [25], we nevertheless tested a panel of primary CNS cell types to assess whether RIPK3 may also influence LGTV tropism. Multistep growth curve analysis in primary CNS cells revealed that pharmacologic inhibition of RIPK3 had no effect on LGTV replication in neurons derived from cerebral cortex (Fig 6D). In contrast, inhibition of RIPK3 significantly enhanced LGTV replication in primary cerebellar granule cell neurons cultures (Fig 6E). This effect was unique to neurons, as GSK 872 treatment had no impact on LGTV replication in primary astrocytes derived from either brain region (Fig 6F–6G). LGTV replication was not detectable by plaque assay in primary microglial cultures; however, we did not observe *Ripk3*-dependent differences in LGTV RNA in primary microglia derived from either brain region (S3A and S3B Fig). Together, these data suggested that the enhanced pathogenesis observed in *Ripk3*^-/-^ mice was due to a specific failure to control infection in neurons of the cerebellum, resulting in enhanced overall CNS viral burden.

### RIPK3 potentiates Type I IFN signaling in cerebellar neurons during LGTV infection

**Fig 7:**
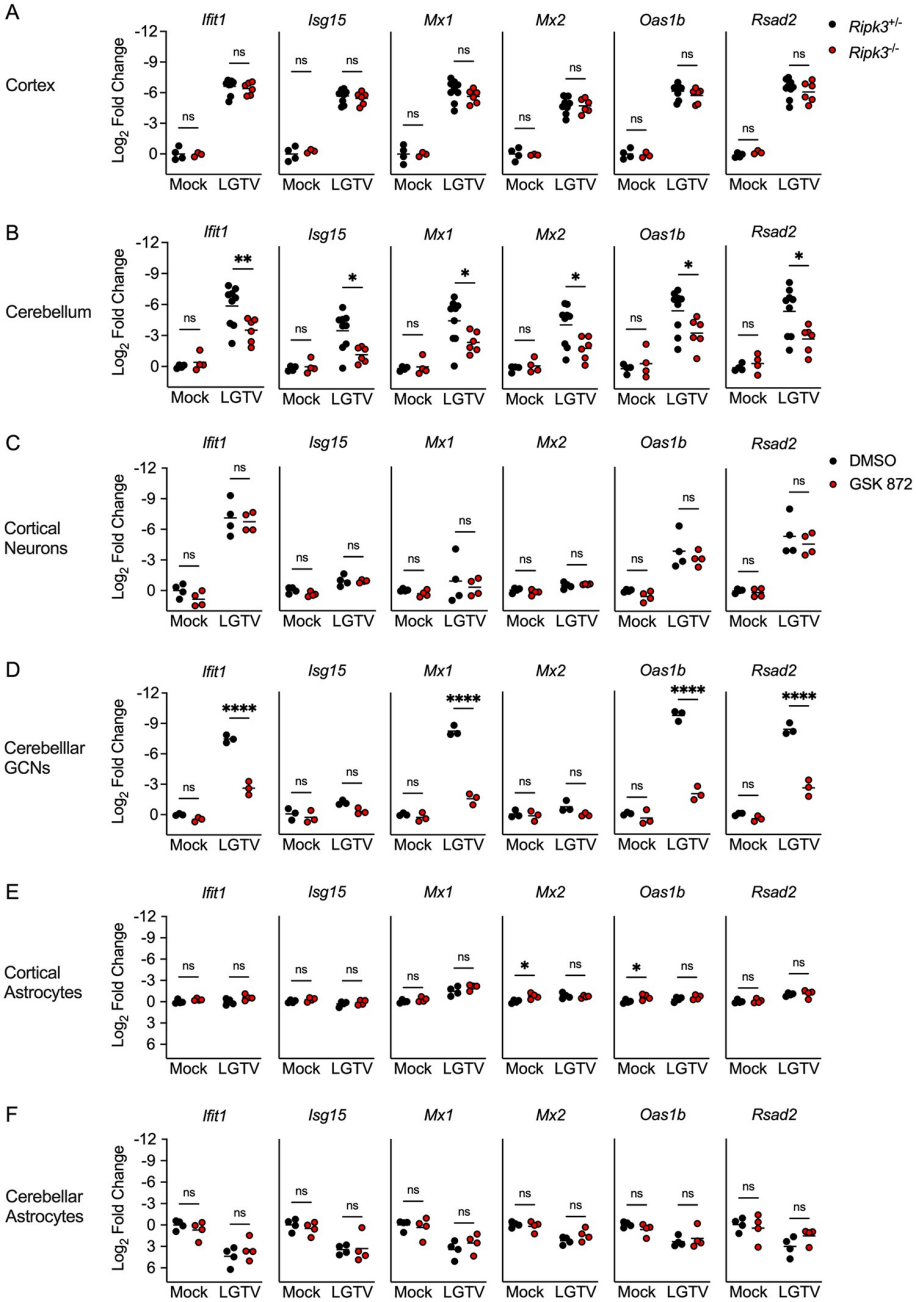
RIPK3 promotes ISG expression in cerebellar granule cell neurons. (A-B) *Ripk3* ^-/-^ and littermate control mice were infected subcutaneously with LGTV TP21. Transcriptional expression of indicated genes was assessed via qRT-PCR in cerebral cortical (A) and cerebellar (B) tissues at 8dpi. (C-D) Transcriptional expression of ISGs in wildtype (C57BL/6J) cultures of primary cortical neurons (C), cerebellar granule cell neurons (D), cortical astrocytes (E), and cerebellar astrocytes (F) following 2-hour pretreatment with GSK 872 or vehicle and 24-hour infection with 0.5 (C-D) or 0.01 (E-F) MOI LGTV TP21, measured via qRT-PCR. ns, not significant. *p<0.05, **p < 0.01, ***p < 0.001, ****p < 0.0001.

Our previous observation of diminished chemokine expression in cerebellar neurons derived from *Ripk3*^-/-^ mice suggested that these cells may exhibit broader deficits in innate immune signaling, resulting in poor control of LGTV replication. Previous work has shown that cerebellar granule cell neurons exhibit enhanced type I IFN signaling compared to cortical neurons [12]. We, therefore, next questioned whether IFN signaling was perturbed in the cerebellum of mice lacking RIPK3 expression. Transcriptional profiling in brain tissues following subcutaneous LGTV infection revealed that, indeed, the cerebella of *Ripk3*^-/-^ mice exhibited diminished expression of many ISGs known to be critical for control of flavivirus replication [30–35], including *Ifit1*, *Isg15*, *Mx1*, *Mx2*, *Oas1b*, and *Rsad2*, while this phenotype was not observed in the cerebral cortex (Fig 7A and 7B). Similar analyses in primary cell cultures confirmed that cerebellar granule cell neurons, but not neurons derived from cerebral cortex, exhibited diminished expression of ISGs when RIPK3 signaling was blocked via GSK 872 treatment (Fig 7C and 7D). In contrast, we observed little to no impact of RIPK3 blockade on ISG expression in astrocytes derived from either brain region (Fig 7E and 7F). Surprisingly, we did observe that lack of *Ripk3* expression diminished ISG expression in primary microglia, although this phenotype occurred in both cerebral cortical and cerebellar microglia cultures (S3B and S3C Fig), suggesting that variations in microglial ISG expression were not the source of regional differences in virologic control in the brain. Differences in ISG expression between regional neuron types were not driven by differential rates of cell death, as pharmacologic blockade of RIPK3 did not impact viability of either neuron type following LGTV infection (S4 Fig). Together, these data demonstrate that RIPK3 signaling is required for the robust induction of type I IFN responses in neurons of the cerebellum, which is required for cell-intrinsic restriction of LGTV replication.

**Fig 8:**
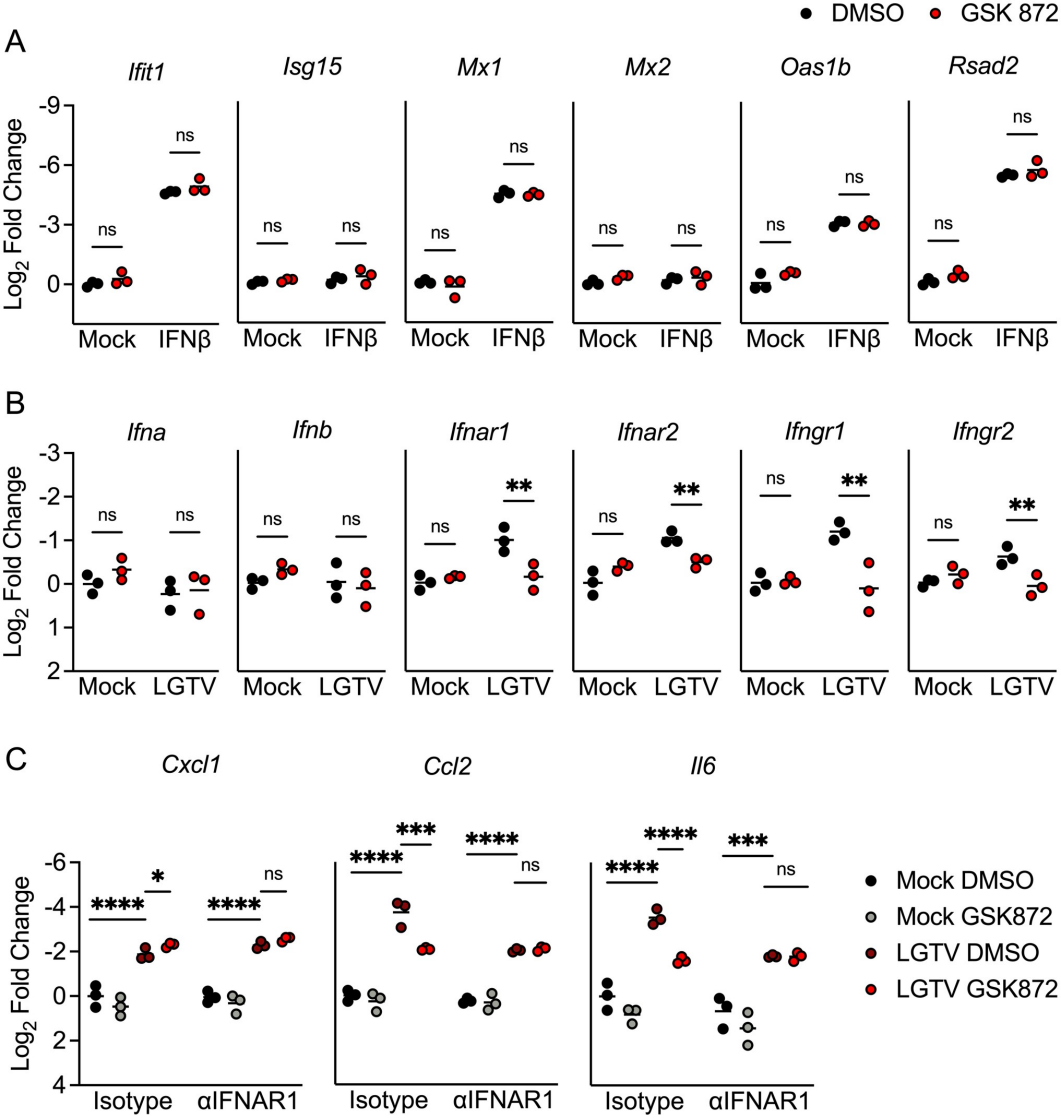
RIPK3 promotes expression of IFN receptors and IFN-dependent inflammatory genes in cerebellar granule cell neurons. A-B) Transcriptional expression of indicated genes in wildtype (C57BL/6J) cultures of cerebellar granule cell neurons in the setting of 2-hour pretreatment with GSK 872 or vehicle followed by 1 hour treatment with 10ng/ml IFNβ (A) or 24-hour infection with 0.5 MOI LGTV TP21 (B). C) Expression of indicated genes in wildtype cerebellar granule cell neurons pretreated for 45 minutes with an anti-IFNAR1 neutralizing antibody or isotype control +/- cotreatment with GSK 872 or vehicle, followed by 24-hour infection with 0.5 MOI LGTV TP21. ns, not significant. *p<0.05, **p < 0.01, ***p < 0.001, ****p < 0.0001.

To better understand the role of RIPK3 signaling in potentiating ISG expression, we next questioned whether RIPK3 acts downstream of IFN receptor signaling. To assess this, we treated neuron cultures with exogenous IFNβ for 1 hour following pretreatment with GSK 872 or vehicle control. As expected, IFNβ treatment resulted in robust induction of multiple ISGs (Figs 8A and S5A). However, pharmacologic blockade of RIPK3 did not impact ISG expression induced by IFNβ treatment in either cerebellar granule cell neurons (Fig 8A) or in cerebral cortical neurons (S5A Fig), suggesting that RIPK3 likely does not act directly downstream of the type I IFN receptor (IFNAR) to modulate gene expression and/or that type I IFN alone is not sufficient to induce RIPK3 activation. We next tested the alternative hypothesis that RIPK3 regulates IFN signaling during LGTV infection by directly influencing the expression of IFN ligands and receptors. Surprisingly, transcriptional analysis revealed that pharmacologic blockade of RIPK3 did not influence the expression of the type I IFN ligands *Ifna* and *Ifnb* in cerebellar granule cell neurons following infection (Fig 8B). In contrast, GSK 872 treatment significantly blunted infection-induced upregulation of IFN receptor subunits, including the type I IFN receptor subunits *Ifnar1* and *Ifnar2*, and the type II IFN receptor subunits *Ifngr1*, and *Infgr2*. Importantly, we did not observe this RIPK3-dependency in IFN receptor expression in cerebral cortical neuron cultures (S5B Fig), suggesting that RIPK3 functions uniquely in cerebellar granule cell neurons to enhance type I IFN signaling during LGTV infection.

To further investigate a role for RIPK3 in IFN-mediated gene expression in cerebellar neurons, we next performed experiments in which we blocked RIPK3 activity with or without simultaneous blockade of type I IFN signaling using a neutralizing antibody against IFNAR1. We reasoned that this paradigm would allow us to assess the differential influence of RIPK3 on IFNAR-dependent and IFNAR-independent gene expression following LGTV infection. Perhaps unsurprisingly, we observed that expression of most ISGs was completely dependent on IFNAR1 signaling, making it difficult to distinguish a specific role for RIPK3 in the absence of intact type I IFN signaling (S5C Fig). We thus identified several alternative inflammatory genes whose expression was either completely (*Cxcl1*) or partially (*Ccl2 and Il6*) independent of IFNAR1 signaling following infection. Notably, pharmacologic blockade of RIPK3 only impacted the IFNAR1-depenent portion of the induced expression of these genes, while having no effect on the IFNAR1-independent portion, as indicated by a lack of effect in αIFNAR1-treated cultures (Fig 8C). Together, these data further support our observation of synergistic signaling between type I IFN and RIPK3 signaling in cerebellar granule cell neurons during LGTV infection.

**Fig 9:**
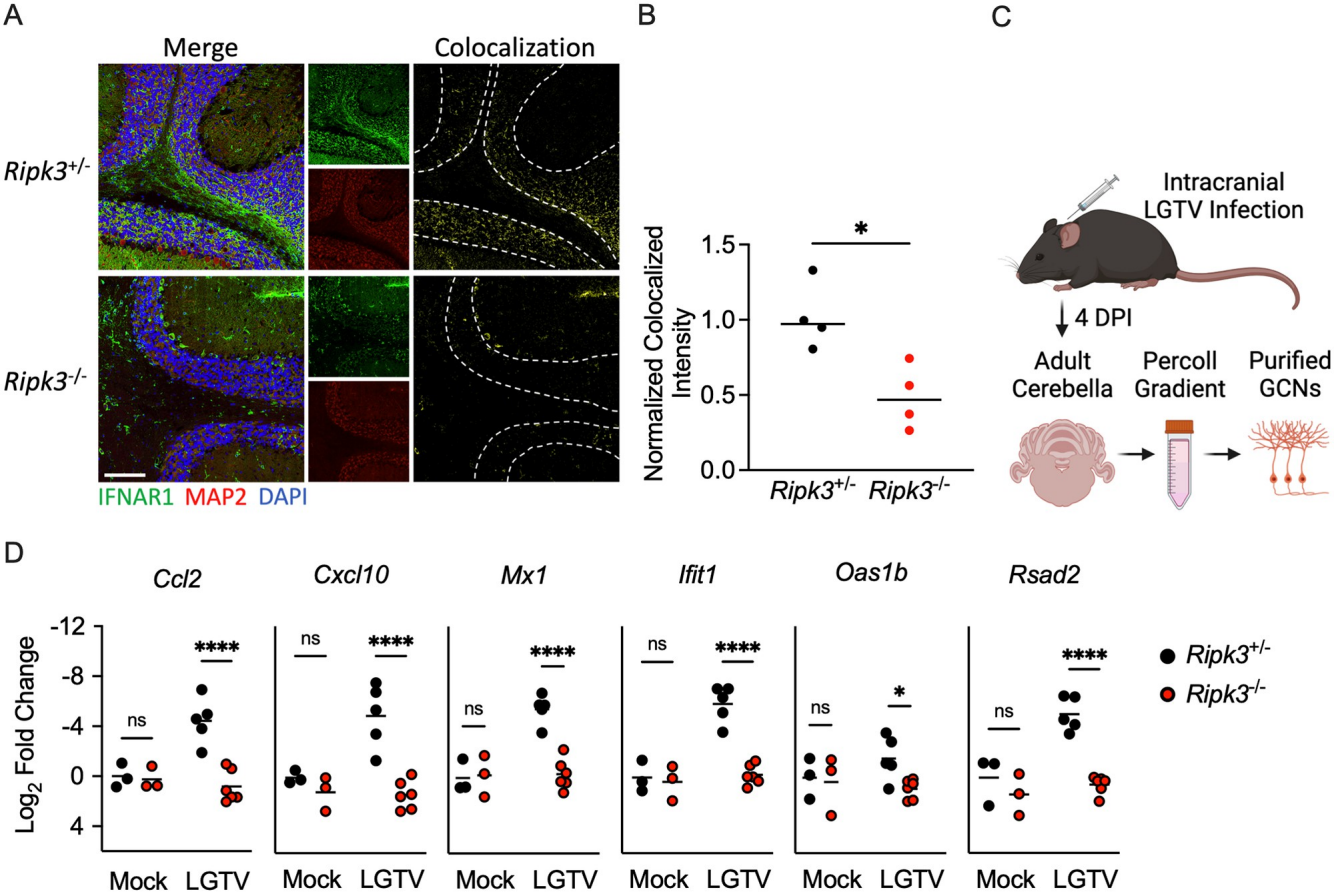
RIPK3 promotes IFN signaling in cerebellar neurons *in vivo*. A-B) Representative images (A) and quantification (B) of IHC analysis of IFNAR1 expression on MAP2 expressing neurons in cerebella of mice at 8 dpi (subcutaneous). Colocalized signaling between IFNAR1 and MAP2 shown in yellow. Traces outline the granule cell layer of the cerebellum. Scale bar = 100um. Quantification represents intensity of colocalized signal normalized to the total size of the granule cell layer in each image. C) Experimental schematic depicting *ex vivo* isolation of adult cerebellar granule cell neurons (GCNs). Image made with BioRender. D) Transcriptional expression of indicated genes in isolated GCNs derived from mice of indicated genotypes at 4 dpi (intracranial). ns, not significant. *p<0.05, ****p < 0.0001. Some figure elements were made with BioRender.

To confirm these observations *in vivo*, we next performed immunohistochemical staining of IFNAR1 in cerebella of mice at 8 days following subcutaneous LGTV challenge. We observed significantly diminished colocalized signal of IFNAR1 on MAP2 expressing neurons within the granule cell layer of the cerebellum in *Ripk3*^-/-^ mice compared to littermate controls (Fig 9A and 9B), confirming that RIPK3 promotes interferon receptor expression in these cells at the protein level. To further confirm whether RIPK3 promoted ISG expression in these cells, we acutely isolated cerebellar granule cell neurons *ex vivo* (Fig 9C) following intracranial LGTV infection. Isolated cells expressed high levels of the glutamate receptor genes *Gria4 and Grid2*, but low to undetectable levels of the glial cell-associated genes *Gfap* and *Aif1*, confirming our isolates were highly enriched for neurons (S6 Fig). Gene expression analysis showed that ISG induction was significantly diminished in cerebellar neurons of *Ripk3*^-/-^ mice compared to controls (Fig 9D), consistent with our previous findings using primary cultures. These data further support our finding that RIPK3 is required for robust induction of interferon signaling in cerebellar neurons during LGTV infection.

## Discussion

Our findings identify a previously unknown function for RIPK3 in the coordination of brain region-specific innate immunity. The study of regional differences in neuroimmune signaling is a growing field, and there is accumulating evidence to suggest that resident neural cells exhibit differential responses to viral infection and cytokine stimulation across distinct anatomical regions of the CNS [36–39]. Neurons and astrocytes in the cerebellum, in particular, have been shown to exhibit higher responsiveness to stimulation by type-I IFN, as well as to express higher basal levels of pathogen sensor molecules compared to other brain regions, suggesting a key evolutionary importance of innate antiviral defense in this tissue [12,13]. This regional difference in type I IFN signaling appears to underlie, at least in part, the relatively lower susceptibility of the cerebellum to flavivirus infection compared to susceptible regions of the forebrain, such as the cerebral cortex and hippocampus. Our findings also underscore the particular importance of IFN signaling in cerebellar granule cell neurons, which account for over 99% of neurons in the cerebellum and more than 50% of the neurons in the entire brain [12,40]. However, the molecular mechanisms that determine the enhanced innate immune signaling observed in the cerebellum remain poorly understood. Our study suggests that RIPK3 signaling is required for the robust induction of ISG expression in cerebellar neurons during LGTV infection, although ongoing work is needed to understand the specific signaling interactions that mediate this effect.

Previous studies have described a highly complex interplay between RIPK3 and type I IFN signaling that varies significantly by cell type and disease model [17–19,41]. It is relatively clear that type I IFN signaling is capable of activating RIPK3 through various mechanisms, resulting in necroptosis and/or necroptosis-independent transcriptional activation [41–44]. However, how RIPK3 operates *upstream* of (or synergistically with) type I IFN signaling to influence expression of ISGs is less clear. We and others have shown that ISG expression is significantly diminished in a variety of settings when RIPK3 signaling is ablated [17,18], including in cerebellar granule cell neurons during LGTV infection in this study. One possible explanation for this effect is RIPK3-mediated activation of NF-κB, a transcription factor strongly associated with RIPK signaling with known roles in potentiating type I IFN signaling and ISG expression [22,45–47]. We and others also previously showed that RIPK3 activation in cortical neurons following ZIKV infection leads to interferon regulatory factor 1 (IRF1) activation, which was required for expression of at least a subset of RIPK3-induced genes in that setting, although this effect is likely indirect, as IRF1 is not a known RIPK3 substrate [17]. Additional work will be needed to fully characterize the regulatory mechanisms that are invoked in the interplay between RIPK3 and type I IFN signaling in the CNS.

Our study also further expands our understanding of the necroptosis-independent functions for RIPK3 signaling in the CNS. Many studies have now firmly established the importance of RIPK3 in promoting host defense through mechanisms independent of its canonical role in necroptosis [16–21]. However, these necroptosis-independent functions appear to vary significantly by disease state, including CNS infection with distinct neuroinvasive flaviviruses [48,49]. We and others previously showed that the primary role for RIPK3 in restricting WNV encephalitis was the induction of chemokine expression and the recruitment of antiviral leukocytes into the infected CNS [16]. Notably, while we did observe RIPK3-mediated chemokine expression in the cerebellum during LGTV infection, this chemokine expression was apparently dispensable for CNS immune cell recruitment. These findings highlight the context-dependent nature by which RIPK3 seems to regulate neuroinflammation. Notably, the RIPK3-dependent neuronal transcriptional program includes both pro- and anti-inflammatory mediators [17,49]. The centrality of IFN signaling in our current study is also notable, as we and others have previously shown that type I IFN suppresses leukocyte infiltration into the CNS during flavivirus encephalitis [13]. Thus, diminished IFN signaling in LGTV-infected *Ripk3*^-/-^ mice may counterbalance the reduced expression of leukocyte chemoattractants in the overall regulation of CNS immune cell recruitment.

Unlike our previous findings using a model of WNV encephalitis [16], the RIPK3-dependent transcriptional activation of antiviral effector genes, including ISGs, was required for cell-intrinsic restriction of LGTV replication in neurons, a phenotype more similar to our findings with ZIKV [17], although we did not observe evidence for a regional specification of this response during ZIKV infection. In contrast to these observations, Bian and colleagues have observed quite distinct phenotypes in a model of JEV encephalitis, wherein both RIPK3 and MLKL appeared to exacerbate rather than restrict disease pathogenesis [50,51]. RIPK3 also appeared to *suppress* rather than promote ISG expression in JEV infected neurons. At present, the factors that determine such distinct outcomes of RIPK3 signaling across this family of closely related viruses and among distinct CNS regions and cell types are mysterious. However, we speculate that the evolutionary pressures that have shaped differential innate immune signaling across distinct regional populations of neurons, such as enhanced pattern recognition receptor signaling in cerebellar granule cell neurons [12], have also likely driven convergent, context-specific functional specialization of *Ripk3*, a gene which exhibits high rates of evolution and strong positive selection across mammalian genera [52]. However, further work is needed to fully define the molecular mechanisms that govern such specializations, as well as to assess whether regional differences in RIPK3 function in the CNS are conserved across species.

## Materials and methods

### Ethics statement

All procedures involving animal care and use were approved by and conducted in accordance with the Rutgers University Institutional Animal Care and Use Committee (IACUC) under protocol number 201900016.

### Mouse lines

*Ripk3*^-/-^ [53] and *Mlkl*^-/-^ [54] mouse lines were bred and housed under specific-pathogen free conditions in Nelson Biological Laboratories at Rutgers University. *Ripk3*^-/-^ mice were generously provided by Genentech, Inc. Wild-type C57BL/6J mice were either obtained commercially (Jackson Laboratories) or bred in-house. Mice used for subcutaneous infections were 5 weeks old; mice used for intracranial infections were 8–15 weeks old.

### Virus and titer determination

Langat virus strain TP21 was used throughout the study. Founder stocks were obtained from the World Reference Center for Emerging Viruses and Arboviruses (WRCEVA). Laboratory stocks were generated using Vero E6 cells (ATCC, #CRL-1586) and frozen at -80°C until needed. Virus titers were determined by plaque assay on Vero E6 cells. Cells were maintained in DMEM (Corning #10-013-CV) supplemented with 10% Heat Inactivated FBS (Gemini Biosciences #100–106), 1% Penicillin–Streptomycin-Glutamine (Gemini Biosciences #400–110), 1% Amphotericin B (Gemini Biosciences #400–104), 1% Non-Essential Amino Acids (Cytiva, #SH30238.01), and 1% HEPES (Cytiva, #SH30237.01). Plaque assay media was composed of 1X EMEM (Lonza # 12-684F) supplemented with 2% Heat Inactivated FBS (Gemini Biosciences #100–106), 1% Penicillin–Streptomycin-Glutamine (Gemini Biosciences, #400–110), 1% Amphotericin B (Gemini Biosciences #400–104), 1% Non-Essential Amino Acids (Cytiva, #SH30238.01), and 1% HEPES (Cytiva, SH30237.01), 0.75% Sodium Bicarbonate (VWR, #BDH9280) and 0.5% Methyl Cellulose (VWR, #K390). Plaque assays were developed at 5dpi by removal of overlay media and staining/fixation using 10% neutral buffered formalin (VWR, #89370) and 0.25% crystal violet (VWR, #0528). Plaque assays were performed by adding 100uL of serially diluted sample for 1 hour at 37°C to 12-well plates containing 200,000 Vero E6 cells per well. Plates were further incubated with plaque assay media at 37°C and 5% CO2 for 5 days. Medium was removed from the wells and replaced with fixative containing crystal violet for approximately 20–30 minutes. Plates were washed repeatedly in H_2_O and allowed to dry before counting visible plaques.

### Mouse infections and tissue harvesting

Isoflurane anesthesia was used for all procedures. Mice were inoculated subcutaneously (50uL) with 3x10^4^ PFU or injected intracranially (10uL) with 50 PFU of LGTV-TP21 using insulin syringes (BD Medical, #BD-329461). At appropriate times post infection, mice underwent cardiac perfusions with 30 mL cold sterile 1X phosphate-buffered saline (PBS). Extracted tissues were weighed and homogenized using 1.0 mm diameter zirconia/silica beads (Biospec Products, #11079110z) in sterile PBS for plaque assay or TRI Reagent (Zymo, #R2050-1) for gene expression analysis. Homogenization was performed in an Omni Beadrupter Elite for 2 sequential cycles of 20 s at a speed of 4 m/s.

### Primary cell infections

Cortical and cerebellar astrocytes and microglia were harvested from P1-P2 pups and cortical neurons were harvested at E13.5-E15.5. Tissues were dissociated using the Neural Dissociation Kit (T) following manufacturer’s instructions (Miltenyi, #130-093-231). Astrocytes were expanded in AM-a medium (ScienCell, #1831) supplemented with 10% FBS in fibronectin-coated cell culture flasks and seeded into plates coated with 20 μg/mL Poly-L-Lysine (Sigma-Aldrich, #9155) before experiments. For microglia cultures, mixed glia were grown in fibronectin-coated cell culture flasks in DMEM supplemented with 10% FBS, 1% PSF and 20 ng/mL recombinant mouse M-CSF (Peprotech, #315–02). Medium was changed every 2–3 days for approximately 1 week, followed by shaking the flasks at 37C at 500rpm for 3–4 hours. Microglia in the supernatant were collected and seeded into PLL-coated cell culture treated plates for subsequent experiments. Neurons were seeded into PLL-coated cell culture treated plates and grown in Neurobasal Plus + B-27 supplement medium (Thermo-Fisher Scientific, #A3582901) prior to use in experiments 7–9 days in vitro (DIV). Mouse cerebellar granule cells from C57BL/6 mice (ScienCell, # M1530-57) were seeded into cell culture treated plates coated with 10 ug/mL Poly-D-Lysine (ThermoFisher, #A3890401) containing prewarmed Neuronal Medium (ScienCell, #1521) following manufacturer recommendations and used for experiments 6 DIV.

Macrophages and dendritic cells were isolated from bone marrow of euthanized mice. Femurs were isolated and bone marrow pushed out using a sterile needle and syringe loaded with RPMI supplemented with 10% FBS, 1% Penicillin–Streptomycin-Glutamine, 1% HEPES, 1% Glutamax (ThermoFisher, #35050061). Bone marrow was plated into non-cell-culture treated 10cm petri dishes in 8mL supplemented RPMI medium containing either 20ng/mL recombinant M-CSF (Peprotech, #315–02) or 20ng/mL recombinant GM-CSF (Peprotech, #315–03) and 20ng/mL IL-4 (Peprotech, #214–14) for differentiation into macrophages or dendritic cells, respectively. Cells were fed with additional medium containing the appropriate cytokines four days later and used for experiments at 6–7 DIV. Cells were seeded into cell-culture treated dishes prior to experimentation. For viral replication determination, all cultures were infected with LGTV TP21 at an MOI of 0.01. For qRT-PCR experiments, cortical and cerebellar neuron cultures were infected at an MOI of 0.5, while astrocyte cultures were infected using an MOI of 0.01. The pharmacologic inhibitor of RIPK3, GSK 872, was added to cultures at 100nM for 2 hours prior to infection or subsequent treatments. Interferon-β was added to neuron cultures at 10ng/mL for 1 hour prior to harvesting cell lysates. IFNAR-1 monoclonal antibody (MAR1-5A3, Leinco Technologies) or isotype control (GIR-208, Leinco Technologies) were added to cultures at 5μg/mL 45 minutes prior to Langat virus infection.

### *Ex vivo* cerebellar granule cell neuron isolation

Adult cerebellar granule cell neurons were isolated using a Percoll gradient as previously described [55], with minor modifications. Adult cerebella were dissociated using an Adult Brain Dissociation Kit (Miltenyi, #130-107-677). Cells were layered over a step gradient of 60% and 35% isotonic Percoll (Cytiva, #17-0891-02) and centrifuged at 2370g for 20 minutes. The middle fraction consisting of granule cells was carefully collected, and acutely isolated cells were immediately lysed for RNA extraction and were not cultured.

### Quantitative real-time PCR

Total RNA from harvested tissues was extracted using Zymo Direct-zol RNA Miniprep kit, as per manufacturer instructions (Zymo, #R2051). Total RNA extraction from cultured cells, cDNA synthesis, and subsequent qRT-PCR were performed as previously described [22,56]. Cycle threshold (CT) values for analyzed genes were normalized to CT values of the housekeeping gene 18 S (CT_Target_ − CT_18S_ = ΔCT). Data from primary cell culture experiments were further normalized to baseline control values (ΔCT_experimental_ − ΔCT_control_ = ΔΔCT (DDCT)). A list of primers used in this study can be found in S1 Table.

### Flow cytometry

The cerebella and cerebral cortices of mouse brains were dissected from freshly perfused mice and placed into tubes containing 1X PBS. Brain tissues were incubated with 10mL buffer containing 0.05% Collagenase Type I (Sigma-Aldrich, #C0130), 10ug/mL DNase I (Sigma-Aldrich, #D4527) and 10mM HEPES (Cytiva, #SH30237.01) in 1X Hanks’ Balanced Salt Solution (VWR, #02-1231-0500) for one hour at room temperature under constant rotation. Brain tissues were transferred to a 70um strainer on 50mL conical tubes and mashed through the strainer using the plunger of 3-5mL syringes. Tissue was separated in 8 mL 37% Isotonic Percoll (Percoll: Cytiva, #17-0891-02; RPMI 1640: Corning, #10-040-CV, supplemented with 5% FBS) by centrifugation at 1200xg for 30 minutes with a slow break. The myelin layer and supernatant were discarded. Leukocytes were incubated in 1X RBC Lysis Buffer (Tonbo Biosciences, #TNB-4300-L100) for 10 minutes at room temperature. Cells were centrifuged and resuspended in FACS buffer composed of 1X PBS, 2% sodium azide and 5% FBS. Samples were transferred into a U-bottomed 96-well plate. Leukocytes were blocked with 2% normal mouse serum and 1% FcX Block (BioLegend, #101320) in FACS buffer for 30 minutes at 4°C prior to being stained with fluorescently conjugated antibodies to CD3e (Biolegend, clone 17A2), CD44 (Biolegend, clone IM7), CD19 (Biolegend, clone 6D5), CD8a (Biolegend, clone 53–6.7), CD4 (Biolegend, clone RM4-5), CD45.2 (Biolegend, clone 104), MHC-II (Biolegend, clone M5/114.15.2), NK1.1 (Biolegend, clone PK136), CD11c (Biolegend, clone N418), F4/80 (Biolegend, clone BM8), CD11b (Biolegend, clone M1/70), Ly6G (Biolegend, clone 1A8), Ly6C (Biolegend, clone HK1.4), CD80 (Biolegend, clone 16-10A1), and Zombie NIR (Biolegend, #423105). Leukocytes were stained for 30 minutes at 4C prior to washing in FACS buffer and fixation with 1% PFA in PBS (ThermoFisher, #J19943-K2). Data collection and analysis were performed using a Cytek Northern Lights Cytometer (Cytek, Fremont, California) and FlowJo software (Treestar). Data were normalized using a standard bead concentration counted by the cytometer with each sample (ThermoFisher, #C36950). Spleens were crushed between two slides, filtered through a 70um cell strainer, and washed with FACS buffer. Isolated splenocytes were incubated with 1X RBC Lysis Buffer as done for leukocytes isolated from the brain prior to blocking and staining.

### IHC

Mice were perfused with ice-cold PBS, followed by perfusion with 30mL cold 4% PFA in PBS (Electron Microscopy Sciences, #15714). Brains were placed in 4% PFA for 24 hours at 4°C, then in PBS containing 0.1% sodium azide at 4°C. Brains were sectioned at 50um using a Compresstome (Precisionary, #VF-510-0Z) and sections stored in PBS with 0.1% sodium azide at 4°C. Sections were incubated in blocking solution consisting of 10% goat serum (Gibco, #16210) and 0.4% Triton X-100 for 3 hours at room temperature under constant rotation. Sections were stained with primary antibodies (mouse-anti-IFNAR-1: 5 ug/mL, Leinco, #I-400; and chicken-anti-MAP2: 1:1000, Invitrogen, #PA1-16751) diluted in blocking solution for 2 days at 4°C. Sections were washed three times with PBS for 5 minutes each at room temperature, followed by incubation with secondary antibodies (goat-anti-chicken IgY conjugated Alexa Fluor 594: 8 ug/mL, Invitrogen, #A32759; and goat-anti-mouse IgG conjugated Alexa Fluor 488: 8 ug/mL, Invitrogen, #A32723) diluted in 10% goat serum in PBS at room temperature for 1 hour. Sections were washed three times and then incubated with DAPI (10 ug/mL, Biotium, #40043) at room temperature for 10 minutes. Sections then mounted onto slides using ProLong Diamond Antifade Mountant (Invitrogen, #P36961). Slides were allowed to dry prior to acquiring images using an Airyscan fluorescent confocal microscope (Carl Zeiss LSM 800).

### In vivo assessment of blood brain barrier permeability

In vivo assessment of blood brain barrier permeability was carried out as described [57]. Mice were injected intraperitoneally with 100uL of 100mg/mL fluorescein sodium salt (Sigma, #F6377) dissolved in sterile 1X PBS. After 45 minutes, blood was collected followed by cardiac perfusion. Tissues were dissected and homogenized in 1X PBS as described above. Serum and supernatant from homogenized tissues were incubated overnight at 4°C with 2% Trichloroacetic acid solution (Sigma, #T0699) at a 1:1 dilution. Precipitated protein was pelleted by 10 minutes of centrifugation at 2,823xg at 4°C. Supernatants were diluted with borate buffer, pH 11 (Sigma, #1094621000) to achieve a neutral pH. Fluorescein emission at 538nm was measured for samples in an optically clear black-walled 96-well plate (Corning, #3904) using a SpectraMax iD3 plate reader (Molecular Devices, San Jose, CA). Tissue fluorescence values were standardized against plasma values for individual mice.

### Statistical analysis

Normally distributed data were analyzed using appropriate parametric tests: two-way analysis of variance (ANOVA) with Sidak’s correction for multiple comparisons and Log-rank (Mantel-Cox) test for survival comparison, both using GraphPad Prism Software v8 (GraphPad Software, San Diego, CA). Chi square tests for comparison of clinical disease signs was performed using Excel v2211 (Microsoft). P < 0.05 was considered statistically significant.

## Supporting information

S1 Fig Neither RIPK3 nor MLKL is required for restriction of LGTV replication in bone marrow-derived macrophages and dendritic cells. (A-B) Multistep growth curve analysis following infection with 0.01 MOI LGTV TP21 in primary macrophages (BMDMs) (A) and dendritic cells (BMDCs) (B) cultured from bone marrow of C57BL/6J (WT), *Ripk3*^-/-^, or *Mlkl*^-/-^ mice. (n = 4) No comparisons are statistically significant.
(TIFF)

S2 Fig RIPK3 promotes protein expression of CXCL10 in the cerebellum and cultured GCNs. A-B) ELISA analysis of CXCL10 abundance in homogenates of cortex (A) or cerebellum (B) derived from mice of indicated genotypes at 8 dpi (footpad). C) CXCL10 ELISA analysis in culture supernatants of GCNs at 24 hpi. ns, not significant. ****p < 0.0001.
(TIFF)

S3 Fig RIPK3 promotes ISG expression independent of CNS region in cultured microglia. A-C) Transcriptional expression of LGTV (A) or indicated genes (B-C) in cultures of cerebral cortical (B) or cerebellar (C) microglia following 24-hour infection with 0.1 MOI LGTV TP21 (B). ns, not significant. *p<0.05, **p < 0.01.
(TIFF)

S4 Fig RIPK3 does not impact cell death in LGTV-infected neurons. A-B) Cell Titer Glo viability assay in wildtype (C57BL/6J) cultures of cerebral cortical neurons or cerebellar granule cell neurons (GCNs) in the setting of 2-hour pretreatment with GSK 872 or vehicle followed by 24-hour infection with 0.5 MOI LGTV TP21.
(TIFF)

S5 Fig RIPK3 does not impact IFN-mediated responses to LGTV in cortical neurons. A-B) Transcriptional expression of indicated genes in wildtype (C57BL/6J) cultures of cerebral cortical neurons in the setting of 2-hour pretreatment with GSK 872 or vehicle followed by 1 hour treatment with 10ng/ml IFNβ (A) or 24-hour infection with 0.5 MOI LGTV TP21 (B). C) Expression of indicated genes in wildtype cerebral cortical neurons pretreated for 45 minutes with an anti-IFNAR1 neutralizing antibody or isotype control +/- cotreatment with GSK 872 or vehicle, followed by 24-hour infection with 0.5 MOI LGTV TP21. ns, not significant. *p<0.05, **p < 0.01, ***p < 0.001, ****p < 0.0001.
(TIFF)

S6 Fig Isolated adult cerebellar granule cell neurons are highly enriched for neuronal genes and not glial genes. Transcriptional expression of indicated genes in isolated GCNs derived from adult WT (C57BL/6J) mice. Values derived from a pool of GCNs isolated from 3 distinct animals.
(TIFF)

S1 Table Primer sequences for qRT-PCR. (DOCX)

## References

[1] Schultz JS, Sparks H, Beckham JD. Arboviral central nervous system infections. Curr Opin Infect Dis. 2021;34(3):264–71. doi: 10.1097/QCO.0000000000000729 33899755

[2] Ruzek D, Avsic Zupanc T, Borde J, Chrdle A, Eyer L, Karganova G, et al. Tick-borne encephalitis in Europe and Russia: Review of pathogenesis, clinical features, therapy, and vaccines. Antiviral Res. 2019;164:23–51. doi: 10.1016/j.antiviral.2019.01.014 30710567

[3] Riccardi N, Antonello RM, Luzzati R, Zajkowska J, Di Bella S, Giacobbe DR. Tick-borne encephalitis in Europe: a brief update on epidemiology, diagnosis, prevention, and treatment. Eur J Intern Med. 2019;62:1–6. doi: 10.1016/j.ejim.2019.01.004 30678880

[4] Abdiyeva K, Turebekov N, Yegemberdiyeva R, Dmitrovskiy A, Yeraliyeva L, Shapiyeva Z, et al. Vectors, molecular epidemiology and phylogeny of TBEV in Kazakhstan and central Asia. Parasit Vectors. 2020;13(1):504. doi: 10.1186/s13071-020-04362-1 33023633 PMC7539389

[5] Beaute J, Spiteri G, Warns-Petit E, Zeller H. Tick-borne encephalitis in Europe, 2012 to 2016. Euro Surveill. 2018;23(45). doi: 10.2807/1560-7917.ES.2018.23.45.1800201 30424829 PMC6234529

[6] Fares M, Cochet-Bernoin M, Gonzalez G, Montero-Menei CN, Blanchet O, Benchoua A, et al. Pathological modeling of TBEV infection reveals differential innate immune responses in human neurons and astrocytes that correlate with their susceptibility to infection. J Neuroinflammation. 2020;17(1):76. doi: 10.1186/s12974-020-01756-x 32127025 PMC7053149

[7] Szretter KJ, Daffis S, Patel J, Suthar MS, Klein RS, Gale M Jr., et al. The innate immune adaptor molecule MyD88 restricts West Nile virus replication and spread in neurons of the central nervous system. J Virol. 2010;84(23):12125–38. doi: 10.1128/JVI.01026-10 20881045 PMC2976388

[8] Iwasaki Y, Zhao JX, Yamamoto T, Konno H. Immunohistochemical demonstration of viral antigens in Japanese encephalitis. Acta Neuropathol. 1986;70(1):79–81. doi: 10.1007/BF00689518 3014801

[9] Klein RS, Lin E, Zhang B, Luster AD, Tollett J, Samuel MA, et al. Neuronal CXCL10 directs CD8+ T-cell recruitment and control of West Nile virus encephalitis. J Virol. 2005;79(17):11457–66. doi: 10.1128/JVI.79.17.11457-11466.2005 16103196 PMC1193600

[10] Lindqvist R, Upadhyay A, Overby AK. Tick-Borne Flaviviruses and the Type I Interferon Response. Viruses. 2018;10(7). doi: 10.3390/v10070340 29933625 PMC6071234

[11] Samuel MA, Diamond MS. Alpha/beta interferon protects against lethal West Nile virus infection by restricting cellular tropism and enhancing neuronal survival. J Virol. 2005;79(21):13350–61. doi: 10.1128/JVI.79.21.13350-13361.2005 16227257 PMC1262587

[12] Cho H, Proll SC, Szretter KJ, Katze MG, Gale M Jr., Diamond MS. Differential innate immune response programs in neuronal subtypes determine susceptibility to infection in the brain by positive-stranded RNA viruses. Nat Med. 2013;19(4):458–64. 23455712 10.1038/nm.3108 PMC3618596

[13] Daniels BP, Jujjavarapu H, Durrant DM, Williams JL, Green RR, White JP, et al. Regional astrocyte IFN signaling restricts pathogenesis during neurotropic viral infection. J Clin Invest. 2017;127(3):843–56. doi: 10.1172/JCI88720 28134626 PMC5330728

[14] Morgan MJ, Kim YS. Roles of RIPK3 in necroptosis, cell signaling, and disease. Exp Mol Med. 2022;54(10):1695–704. doi: 10.1038/s12276-022-00868-z 36224345 PMC9636380

[15] Samson AL, Zhang Y, Geoghegan ND, Gavin XJ, Davies KA, Mlodzianoski MJ, et al. MLKL trafficking and accumulation at the plasma membrane control the kinetics and threshold for necroptosis. Nat Commun. 2020;11(1):3151. doi: 10.1038/s41467-020-16887-1 32561730 PMC7305196

[16] Daniels BP, Snyder AG, Olsen TM, Orozco S, Oguin TH 3rd, Tait SWG, et al. RIPK3 Restricts Viral Pathogenesis via Cell Death-Independent Neuroinflammation. Cell. 2017;169(2):301–13 e11. doi: 10.1016/j.cell.2017.03.011 28366204 PMC5405738

[17] Daniels BP, Kofman SB, Smith JR, Norris GT, Snyder AG, Kolb JP, et al. The Nucleotide Sensor ZBP1 and Kinase RIPK3 Induce the Enzyme IRG1 to Promote an Antiviral Metabolic State in Neurons. Immunity. 2019;50(1):64–76 e4. doi: 10.1016/j.immuni.2018.11.017 30635240 PMC6342485

[18] Downey J, Pernet E, Coulombe F, Allard B, Meunier I, Jaworska J, et al. RIPK3 interacts with MAVS to regulate type I IFN-mediated immunity to Influenza A virus infection. PLoS Pathog. 2017;13(4):e1006326. doi: 10.1371/journal.ppat.1006326 28410401 PMC5406035

[19] Saleh D, Najjar M, Zelic M, Shah S, Nogusa S, Polykratis A, et al. Kinase Activities of RIPK1 and RIPK3 Can Direct IFN-beta Synthesis Induced by Lipopolysaccharide. J Immunol. 2017;198(11):4435–47. 28461567 10.4049/jimmunol.1601717 PMC5471631

[20] Guo H, Koehler HS, Mocarski ES, Dix RD. RIPK3 and caspase 8 collaborate to limit herpes simplex encephalitis. PLoS Pathog. 2022;18(9):e1010857. doi: 10.1371/journal.ppat.1010857 36121858 PMC9521923

[21] Peng R, Wang CK, Wang-Kan X, Idorn M, Kjaer M, Zhou FY, et al. Human ZBP1 induces cell death-independent inflammatory signaling via RIPK3 and RIPK1. EMBO Rep. 2022;23(12):e55839. doi: 10.15252/embr.202255839 36268590 PMC9724671

[22] Chou TW, Chang NP, Krishnagiri M, Patel AP, Lindman M, Angel JP, et al. Fibrillar alpha-synuclein induces neurotoxic astrocyte activation via RIP kinase signaling and NF-kappaB. Cell Death Dis. 2021;12(8):756. 34333522 10.1038/s41419-021-04049-0 PMC8325686

[23] Li S, Zhang Y, Guan Z, Ye M, Li H, You M, et al. SARS-CoV-2 Z-RNA activates the ZBP1-RIPK3 pathway to promote virus-induced inflammatory responses. Cell Res. 2023. doi: 10.1038/s41422-022-00775-y 36650286 PMC9844202

[24] Najjar M, Saleh D, Zelic M, Nogusa S, Shah S, Tai A, et al. RIPK1 and RIPK3 Kinases Promote Cell-Death-Independent Inflammation by Toll-like Receptor 4. Immunity. 2016;45(1):46–59. doi: 10.1016/j.immuni.2016.06.007 27396959 PMC4956514

[25] Baker DG, Woods TA, Butchi NB, Morgan TM, Taylor RT, Sunyakumthorn P, et al. Toll-like receptor 7 suppresses virus replication in neurons but does not affect viral pathogenesis in a mouse model of Langat virus infection. J Gen Virol. 2013;94(Pt 2):336–47. doi: 10.1099/vir.0.043984-0 23136362 PMC3709612

[26] Michlmayr D, Bardina SV, Rodriguez CA, Pletnev AG, Lim JK. Dual Function of Ccr5 during Langat Virus Encephalitis: Reduction in Neutrophil-Mediated Central Nervous System Inflammation and Increase in T Cell-Mediated Viral Clearance. J Immunol. 2016;196(11):4622–31. doi: 10.4049/jimmunol.1502452 27183602 PMC4973474

[27] Hubbard NW, Ames JM, Maurano M, Chu LH, Somfleth KY, Gokhale NS, et al. ADAR1 mutation causes ZBP1-dependent immunopathology. Nature. 2022;607(7920):769–75. doi: 10.1038/s41586-022-04896-7 35859177 PMC9339495

[28] Xie Y, Zhao Y, Shi L, Li W, Chen K, Li M, et al. Gut epithelial TSC1/mTOR controls RIPK3-dependent necroptosis in intestinal inflammation and cancer. J Clin Invest. 2020;130(4):2111–28. doi: 10.1172/JCI133264 31961824 PMC7108921

[29] Sai K, Parsons C, House JS, Kathariou S, Ninomiya-Tsuji J. Necroptosis mediators RIPK3 and MLKL suppress intracellular Listeria replication independently of host cell killing. J Cell Biol. 2019;218(6):1994–2005. doi: 10.1083/jcb.201810014 30975711 PMC6548127

[30] Kimura T, Katoh H, Kayama H, Saiga H, Okuyama M, Okamoto T, et al. Ifit1 inhibits Japanese encephalitis virus replication through binding to 5’ capped 2’-O unmethylated RNA. J Virol. 2013;87(18):9997–10003. doi: 10.1128/JVI.00883-13 23824812 PMC3754022

[31] Szretter KJ, Daniels BP, Cho H, Gainey MD, Yokoyama WM, Gale M Jr., et al. 2’-O methylation of the viral mRNA cap by West Nile virus evades ifit1-dependent and -independent mechanisms of host restriction in vivo. PLoS Pathog. 2012;8(5):e1002698. doi: 10.1371/journal.ppat.1002698 22589727 PMC3349756

[32] Dai J, Pan W, Wang P. ISG15 facilitates cellular antiviral response to dengue and west nile virus infection in vitro. Virol J. 2011;8:468. doi: 10.1186/1743-422X-8-468 21992229 PMC3215395

[33] Singh PK, Singh S, Farr D, Kumar A. Interferon-stimulated gene 15 (ISG15) restricts Zika virus replication in primary human corneal epithelial cells. Ocul Surf. 2019;17(3):551–9. doi: 10.1016/j.jtos.2019.03.006 30905842 PMC6708474

[34] Bigham AW, Buckingham KJ, Husain S, Emond MJ, Bofferding KM, Gildersleeve H, et al. Host genetic risk factors for West Nile virus infection and disease progression. PLoS One. 2011;6(9):e24745. doi: 10.1371/journal.pone.0024745 21935451 PMC3174177

[35] Vonderstein K, Nilsson E, Hubel P, Nygard Skalman L, Upadhyay A, Pasto J, et al. Viperin Targets Flavivirus Virulence by Inducing Assembly of Noninfectious Capsid Particles. J Virol. 2018;92(1). doi: 10.1128/JVI.01751-17 29046456 PMC5730767

[36] Williams JL, Manivasagam S, Smith BC, Sim J, Vollmer LL, Daniels BP, et al. Astrocyte-T cell crosstalk regulates region-specific neuroinflammation. Glia. 2020;68(7):1361–74. doi: 10.1002/glia.23783 31961459 PMC7317491

[37] Simmons SB, Liggitt D, Goverman JM. Cytokine-regulated neutrophil recruitment is required for brain but not spinal cord inflammation during experimental autoimmune encephalomyelitis. J Immunol. 2014;193(2):555–63. doi: 10.4049/jimmunol.1400807 24913979 PMC4123857

[38] Pierson ER, Goverman JM. GM-CSF is not essential for experimental autoimmune encephalomyelitis but promotes brain-targeted disease. JCI Insight. 2017;2(7):e92362. doi: 10.1172/jci.insight.92362 28405624 PMC5374070

[39] Durrant DM, Daniels BP, Pasieka T, Dorsey D, Klein RS. CCR5 limits cortical viral loads during West Nile virus infection of the central nervous system. J Neuroinflammation. 2015;12:233. doi: 10.1186/s12974-015-0447-9 26667390 PMC4678669

[40] Rhee JK, Park H, Kim T, Yamamoto Y, Tanaka-Yamamoto K. Projection-dependent heterogeneity of cerebellar granule cell calcium responses. Mol Brain. 2021;14(1):63. doi: 10.1186/s13041-021-00773-y 33789707 PMC8011397

[41] McComb S, Cessford E, Alturki NA, Joseph J, Shutinoski B, Startek JB, et al. Type-I interferon signaling through ISGF3 complex is required for sustained Rip3 activation and necroptosis in macrophages. Proc Natl Acad Sci U S A. 2014;111(31):E3206–13. doi: 10.1073/pnas.1407068111 25049377 PMC4128105

[42] Brault M, Olsen TM, Martinez J, Stetson DB, Oberst A. Intracellular Nucleic Acid Sensing Triggers Necroptosis through Synergistic Type I IFN and TNF Signaling. J Immunol. 2018;200(8):2748–56. doi: 10.4049/jimmunol.1701492 29540580 PMC5893403

[43] Ingram JP, Thapa RJ, Fisher A, Tummers B, Zhang T, Yin C, et al. ZBP1/DAI Drives RIPK3-Mediated Cell Death Induced by IFNs in the Absence of RIPK1. J Immunol. 2019;203(5):1348–55. doi: 10.4049/jimmunol.1900216 31358656 PMC6702065

[44] Lee SA, Chang LC, Jung W, Bowman JW, Kim D, Chen W, et al. OASL phase condensation induces amyloid-like fibrillation of RIPK3 to promote virus-induced necroptosis. Nat Cell Biol. 2023. doi: 10.1038/s41556-022-01039-y 36604592 PMC9859756

[45] Yatim N, Jusforgues-Saklani H, Orozco S, Schulz O, Barreira da Silva R, Reis e Sousa C, et al. RIPK1 and NF-kappaB signaling in dying cells determines cross-priming of CD8(+) T cells. Science. 2015;350(6258):328–34. 26405229 10.1126/science.aad0395 PMC4651449

[46] Snyder AG, Hubbard NW, Messmer MN, Kofman SB, Hagan CE, Orozco SL, et al. Intratumoral activation of the necroptotic pathway components RIPK1 and RIPK3 potentiates antitumor immunity. Sci Immunol. 2019;4(36). doi: 10.1126/sciimmunol.aaw2004 31227597 PMC6831211

[47] Wegner KW, Saleh D, Degterev A. Complex Pathologic Roles of RIPK1 and RIPK3: Moving Beyond Necroptosis. Trends Pharmacol Sci. 2017;38(3):202–25. doi: 10.1016/j.tips.2016.12.005 28126382 PMC5325808

[48] Angel JP, Daniels BP. Paradoxical roles for programmed cell death signaling during viral infection of the central nervous system. Curr Opin Neurobiol. 2022;77:102629. doi: 10.1016/j.conb.2022.102629 36162201 PMC10754211

[49] Daniels BP, Oberst A. Outcomes of RIP Kinase Signaling During Neuroinvasive Viral Infection. Curr Top Microbiol Immunol. 2020. doi: 10.1007/82_2020_204 32253569 PMC7781604

[50] Bian P, Ye C, Zheng X, Luo C, Yang J, Li M, et al. RIPK3 Promotes JEV Replication in Neurons via Downregulation of IFI44L. Front Microbiol. 2020;11:368. doi: 10.3389/fmicb.2020.00368 32265853 PMC7105639

[51] Bian P, Zheng X, Wei L, Ye C, Fan H, Cai Y, et al. MLKL Mediated Necroptosis Accelerates JEV-Induced Neuroinflammation in Mice. Front Microbiol. 2017;8:303. doi: 10.3389/fmicb.2017.00303 28293227 PMC5328978

[52] Palmer SN, Chappidi S, Pinkham C, Hancks DC. Evolutionary Profile for (Host and Viral) MLKL Indicates Its Activities as a Battlefront for Extensive Counteradaptation. Mol Biol Evol. 2021;38(12):5405–22. doi: 10.1093/molbev/msab256 34436583 PMC8662602

[53] Newton K, Sun X, Dixit VM. Kinase RIP3 is dispensable for normal NF-kappa Bs, signaling by the B-cell and T-cell receptors, tumor necrosis factor receptor 1, and Toll-like receptors 2 and 4. Mol Cell Biol. 2004;24(4):1464–9. doi: 10.1128/MCB.24.4.1464-1469.2004 14749364 PMC344190

[54] Murphy JM, Czabotar PE, Hildebrand JM, Lucet IS, Zhang JG, Alvarez-Diaz S, et al. The pseudokinase MLKL mediates necroptosis via a molecular switch mechanism. Immunity. 2013;39(3):443–53. doi: 10.1016/j.immuni.2013.06.018 24012422

[55] Klein RS, Rubin JB, Gibson HD, DeHaan EN, Alvarez-Hernandez X, Segal RA, et al. SDF-1 alpha induces chemotaxis and enhances Sonic hedgehog-induced proliferation of cerebellar granule cells. Development. 2001;128(11):1971–81. doi: 10.1242/dev.128.11.1971 11493520

[56] Kung PL, Chou TW, Lindman M, Chang NP, Estevez I, Buckley BD, et al. Zika virus-induced TNF-alpha signaling dysregulates expression of neurologic genes associated with psychiatric disorders. J Neuroinflammation. 2022;19(1):100. 35462541 10.1186/s12974-022-02460-8 PMC9036774

[57] Daniels BP, Holman DW, Cruz-Orengo L, Jujjavarapu H, Durrant DM, Klein RS. Viral pathogen-associated molecular patterns regulate blood-brain barrier integrity via competing innate cytokine signals. mBio. 2014;5(5):e01476–14. doi: 10.1128/mBio.01476-14 25161189 PMC4173776

