# Supplementary figures and images for: RIPK3 promotes brain region-specific interferon signaling and restriction of tick-borne flavivirus infection
Source: PLoS Pathog. 2023 Nov 27;19(11):e1011813. doi: 10.1371/journal.ppat.1011813 (PMC10703404; doi:10.1371/journal.ppat.1011813)

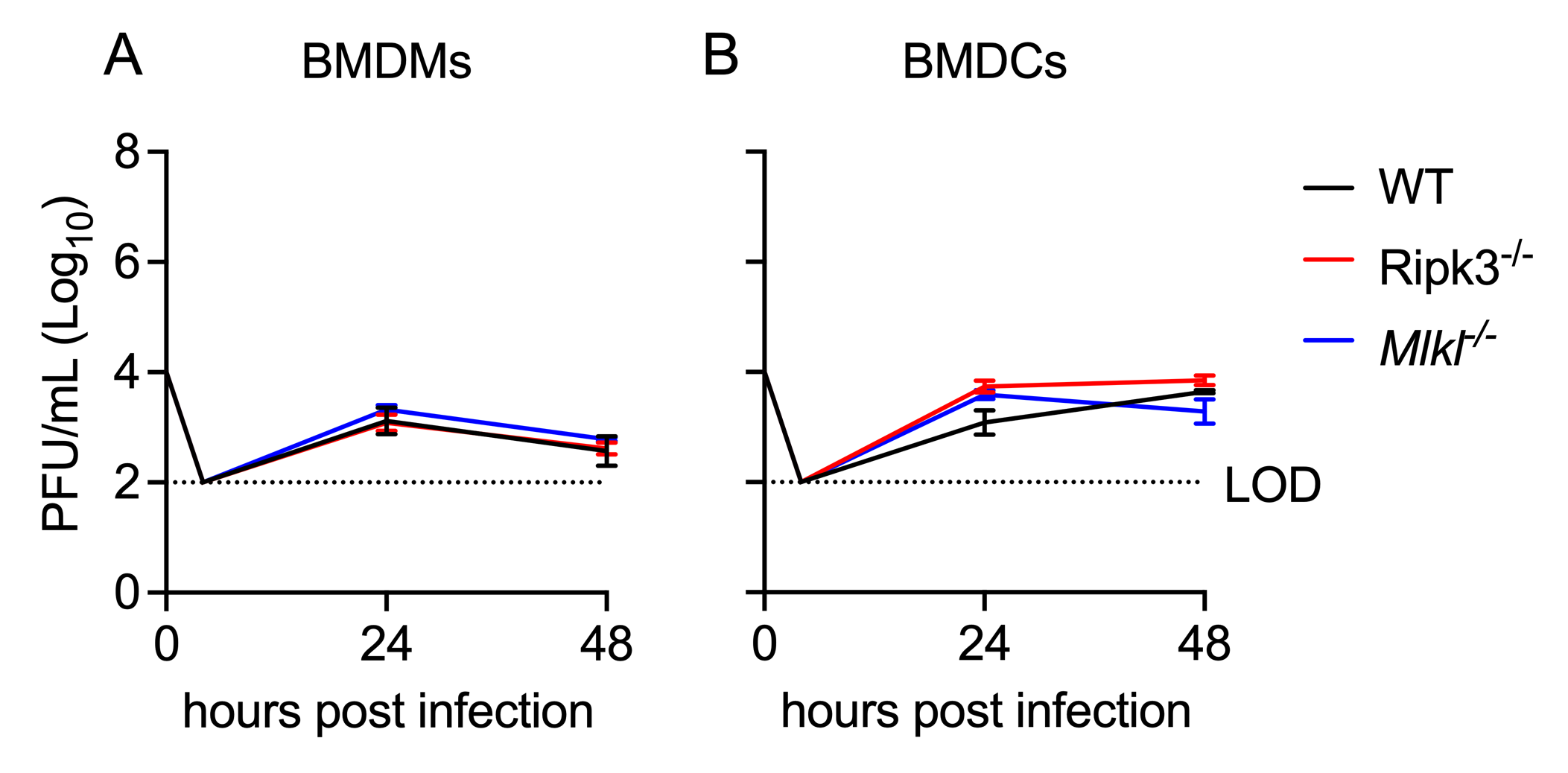

Supplement: S1 Fig — (A-B) Multistep growth curve analysis following infection with 0.01 MOI LGTV TP21 in primary macrophages (BMDMs) (A) and dendritic cells (BMDCs) (B) cultured from bone marrow of C57BL/6J (WT), Ripk3-/-, or Mlkl-/- mice. (n = 4) No comparisons are statistically significant. (TIFF) [file ppat.1011813.s001.tiff]

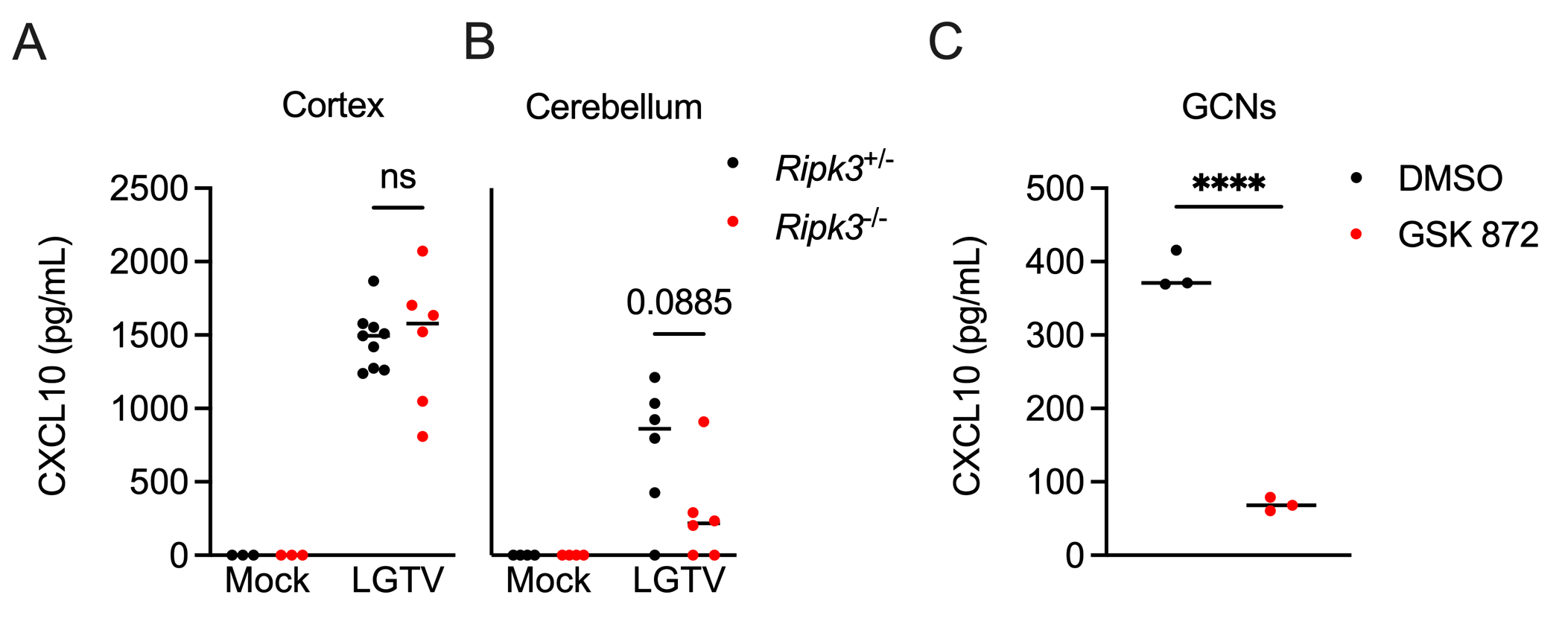

Supplement: S2 Fig — A-B) ELISA analysis of CXCL10 abundance in homogenates of cortex (A) or cerebellum (B) derived from mice of indicated genotypes at 8 dpi (footpad). C) CXCL10 ELISA analysis in culture supernatants of GCNs at 24 hpi. ns, not significant. ****p < 0.0001. (TIFF) [file ppat.1011813.s002.tiff]

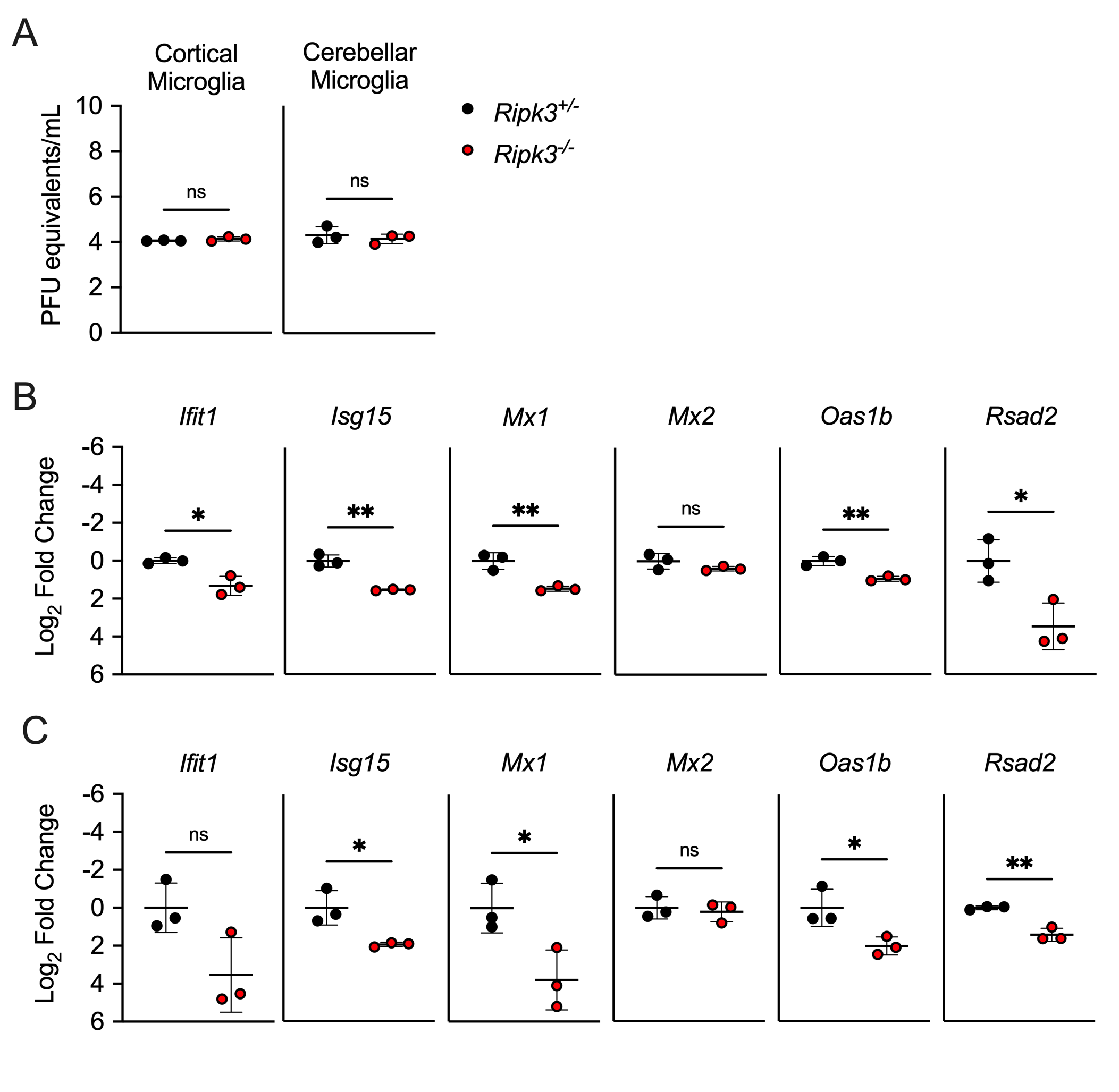

Supplement: S3 Fig — A-C) Transcriptional expression of LGTV (A) or indicated genes (B-C) in cultures of cerebral cortical (B) or cerebellar (C) microglia following 24-hour infection with 0.1 MOI LGTV TP21 (B). ns, not significant. *p<0.05, **p < 0.01. (TIFF) [file ppat.1011813.s003.tiff]

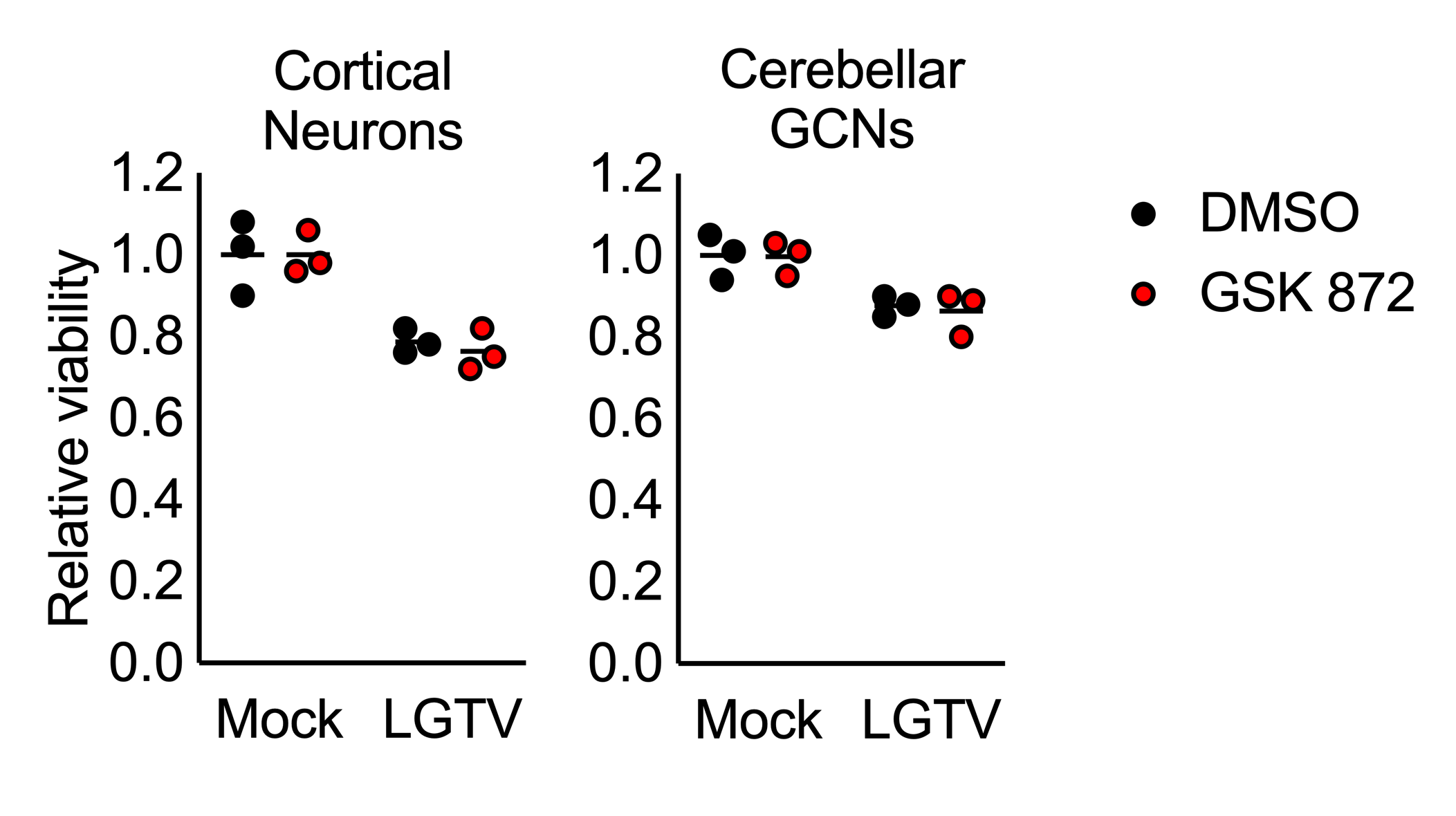

Supplement: S4 Fig — A-B) Cell Titer Glo viability assay in wildtype (C57BL/6J) cultures of cerebral cortical neurons or cerebellar granule cell neurons (GCNs) in the setting of 2-hour pretreatment with GSK 872 or vehicle followed by 24-hour infection with 0.5 MOI LGTV TP21. (TIFF) [file ppat.1011813.s004.tiff]

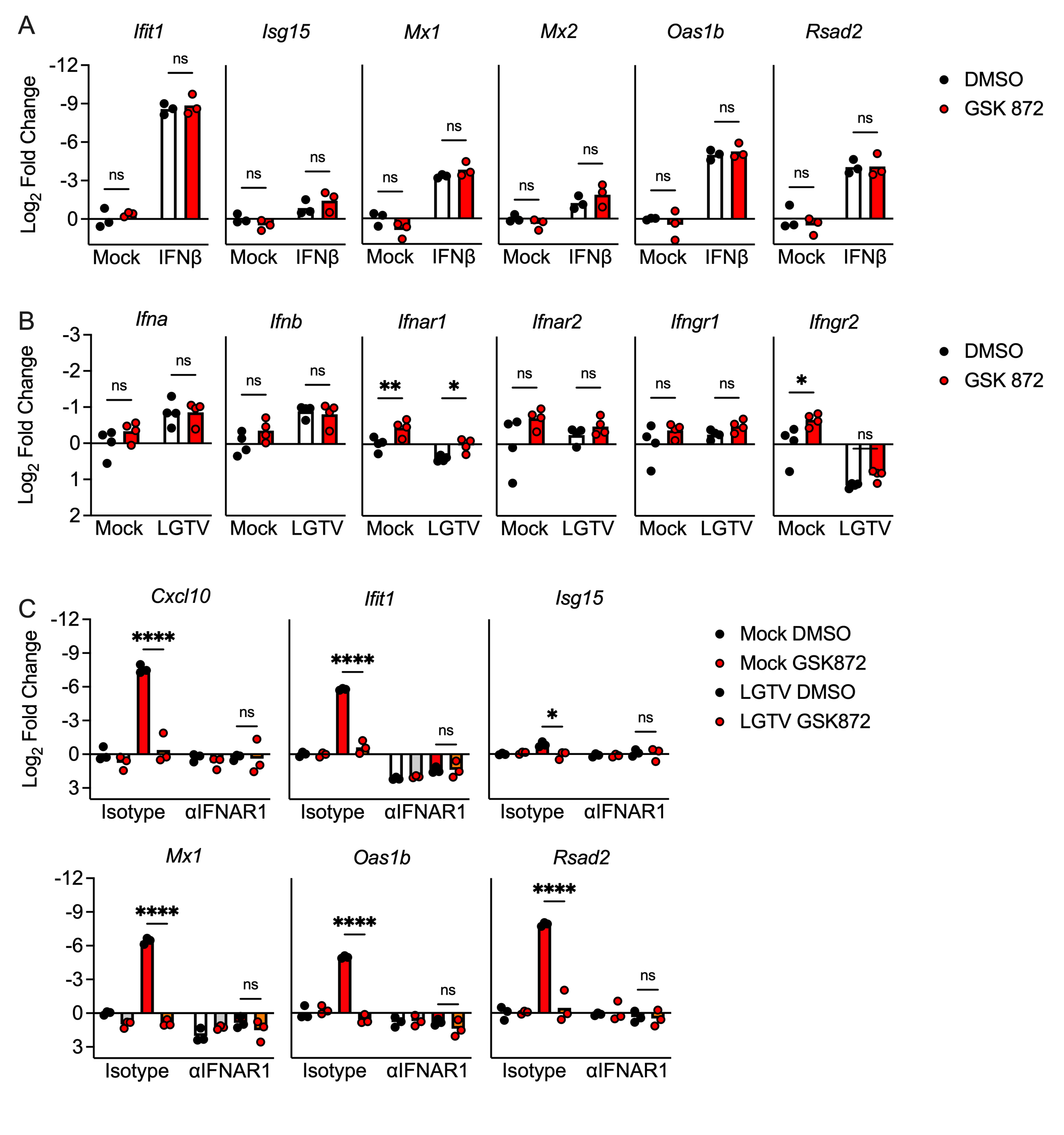

Supplement: S5 Fig — A-B) Transcriptional expression of indicated genes in wildtype (C57BL/6J) cultures of cerebral cortical neurons in the setting of 2-hour pretreatment with GSK 872 or vehicle followed by 1 hour treatment with 10ng/ml IFNβ (A) or 24-hour infection with 0.5 MOI LGTV TP21 (B). C) Expression of indicated genes in wildtype cerebral cortical neurons pretreated for 45 minutes with an anti-IFNAR1 neutralizing antibody or isotype control +/- cotreatment with GSK 872 or vehicle, followed by 24-hour infection with 0.5 MOI LGTV TP21. ns, not significant. *p<0.05, **p < 0.01, ***p < 0.001, ****p < 0.0001. (TIFF) [file ppat.1011813.s005.tiff]

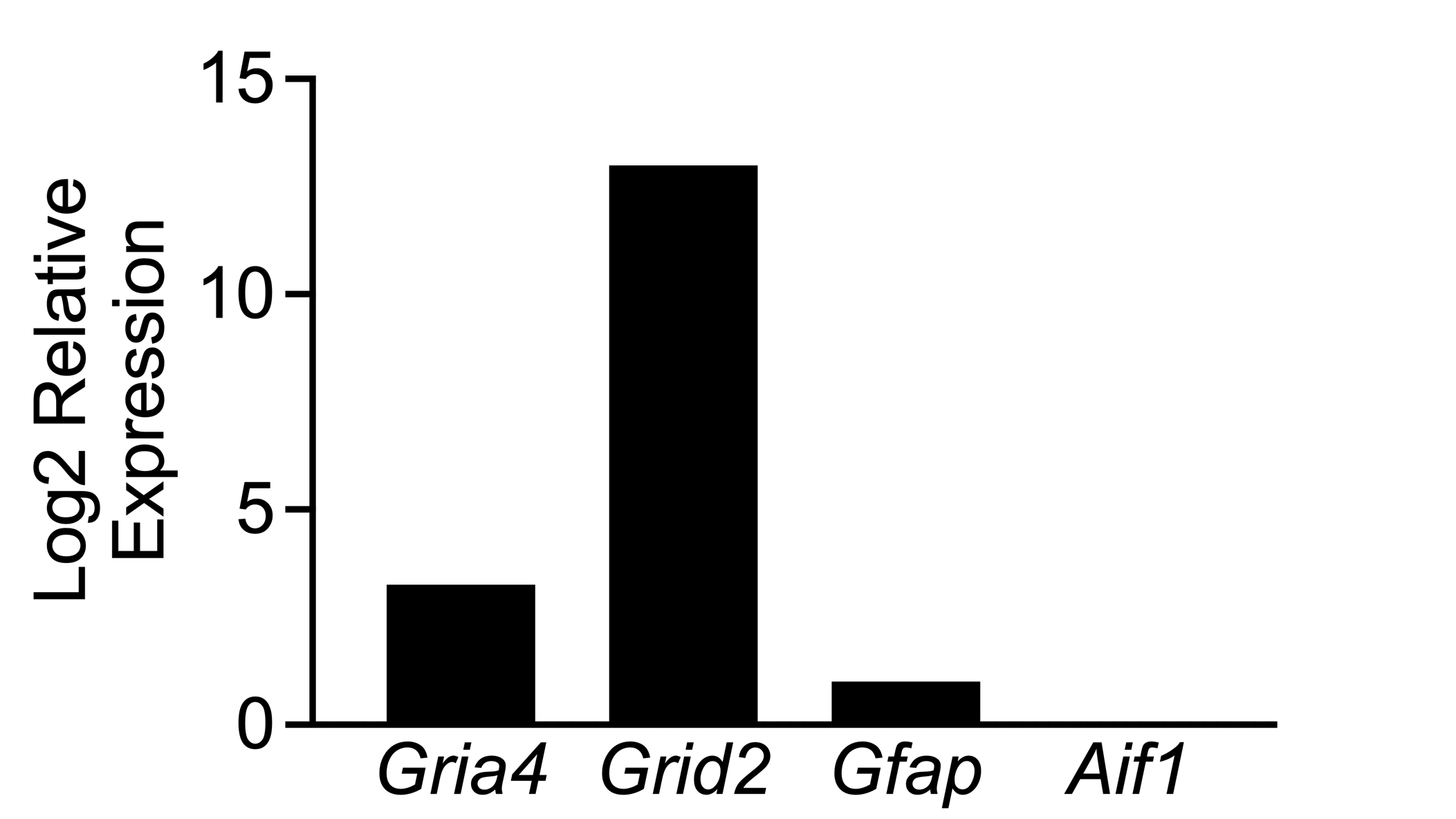

Supplement: S6 Fig — Transcriptional expression of indicated genes in isolated GCNs derived from adult WT (C57BL/6J) mice. Values derived from a pool of GCNs isolated from 3 distinct animals. (TIFF) [file ppat.1011813.s006.tiff]
