# Supplementary material for: RIPK3 promotes brain region-specific interferon signaling and restriction of tick-borne flavivirus infection
Source: PLoS Pathog. 2023 Nov 27;19(11):e1011813. doi: 10.1371/journal.ppat.1011813 (PMC10703404; doi:10.1371/journal.ppat.1011813)
Supplement: S1 Table — (DOCX) [file ppat.1011813.s007.docx]

| **Supplemental Table 1: Primer sequences for qRT-PCR** | | |
| --- | --- | --- |
| Gene | Direction | Sequence (5'-3') |
| *18S* | Forward | CTTAGAGGGACAAGTGGCG |
| *18S* | Reverse | ACGCTGAGCCAGTCAGTGTA |
| *Ccl2* | Forward | TGG CTC AGC CAG ATG CAG T |
| *Ccl2* | Reverse | TTG GGA TCA TCT TGC TGG TG |
| *Ccl3* | Forward | CCA AGT CTT CTC AGC GCC AT |
| *Ccl3* | Reverse | TCC GGC TGT AGG AGA AGC AG |
| *Ccl4* | Forward | TCT TGC TCG TGG CTG CCT |
| *Ccl4* | Reverse | GGG AGG GTC AGA GCC CA |
| *Ccl5* | Forward | CCTGCTGCTTTGCCTACCTCTC |
| *Ccl5* | Reverse | ACACACTTGGCGGTTCCTTCGA |
| *Cxcl1* | Forward | ATCCAGAGCTTGAAGGTGTTG |
| *Cxcl1* | Reverse | GTCTGTCTTCTTTCTCCGTTACTT |
| *Cxcl10* | Forward | CCCACGTGTTGAGATCATTG |
| *Cxcl10* | Reverse | CACTGGGTAAAGGGGAGTGA |
| *Ifit1* | Forward | CCCAGAGAACAGCTACCACC |
| *Ifit1* | Reverse | TGTGAAGTGACATCTCAGCTGA |
| *Ifna* | Forward | CTTCCACAGGATCACTGTGTACCT |
| *Ifna* | Reverse | TTCTGCTCTGACCACCTCCC |
| *Ifnar1* | Forward | TGTGCTTCCCACCACTCAAG |
| *Ifnar1* | Reverse | AGGCGCGTGCTTTACTTCTA |
| *Ifnar2* | Forward | AGACTCTTCGGGTCGCGG |
| *Ifnar2* | Reverse | GTTTTTCTGCTCTCACACCTGA |
| *Ifnb* | Forward | CTGGAGCAGCTGAATGGAAAG |
| *Ifnb* | Reverse | CTTCTCCGTCATCTCCATAGGG |
| *Ifng* | Forward | CCTCATGGCTGTTTCTGGCT |
| *Ifng* | Reverse | TCATGTCACCATCCTTTTGCC |
| *Ifngr1* | Forward | GTGGAGCTTTGACGAGCACT |
| *Ifngr1* | Reverse | TCAGTCCAGGAACCCGAATA |
| *Ifngr2* | Forward | CGTCCTCGCCAGACTCGTT |
| *Ifngr2* | Reverse | AGCAACCTATGCCAAGAGCC |
| *Il6* | Forward | ACACATGTTCTCTGGGAAATCGT |
| *Il6* | Reverse | AAGTGCATCATCGTTGTTCATACA |
| *Isg15* | Forward | TGCCTGCAGTTCTGTACCAC |
| *Isg15* | Reverse | AGTGCTCCAGGACGGTCTTA |
| *Mx1* | Forward | ACTATGAGGAGAAGGTGCGG |
| *Mx1* | Reverse | ACTTTGCCTCTCCACTCCTC |
| *Mx2* | Forward | GCCACGTTCCCTTGATCATC |
| *Mx2* | Reverse | AGCCAGCTTAACCAGGGAAT |
| *Oas1b* | Forward | TTCTACGCCAATCTCATCAGTG |
| *Oas1b* | Reverse | GGTCCCCCAGCTTCTCCTTAC |
| *Rsad2* | Forward | TCAAAAGCTGAGGAGGTGGTG |
| *Rsad2* | Reverse | TAGGAGGCACTGGAAAACCTTC |
